# In Silico Identification of Putative Allosteric Pockets and Inhibitors for the KRASG13D-SOS1 Complex in Cancer Therapy

**DOI:** 10.3390/ijms26073293

**Published:** 2025-04-02

**Authors:** Zehra Sarica, Ozge Kurkcuoglu, Fethiye Aylin Sungur

**Affiliations:** 1 Computational Science and Engineering Division, Informatics Institute, Istanbul Technical University, Istanbul 34469, Türkiye; sarica16@itu.edu.tr; 2 Department of Chemical Engineering, Istanbul Technical University, Istanbul 34469, Türkiye

**Keywords:** KRASG13D-SOS1 complex, allostery, molecular docking, molecular dynamics simulations, natural compounds

## Abstract

RAS mutations occur in about 30% of human cancers, leading to enhanced RAS signaling and tumor growth. KRAS is the most commonly mutated oncogene in human tumors, especially lung, pancreatic, and colorectal cancers. Direct targeting of KRAS is difficult due to its highly conserved sequence; but, its complex with the guanine nucleotide exchange factor Son of Sevenless (SOS) 1 promises an attractive target for inhibiting RAS-mediated signaling. Here, we first revealed putative allosteric binding sites of the SOS1, KRASG12C-SOS1 complex, and the ternary KRASG13D-SOS1 complex structures using two network-based models, the essential site scanning analysis and the residue interaction network model. The results enabled us to identify two new putative allosteric pockets for the ternary KRASG13D-SOS1 complex. These were then screened together with the known ligand binding site against the natural compounds in the InterBioScreen (IBS) database using the Glide software package developed by Schrödinger, Inc. The docking poses of seven hit compounds were assessed using 400 ns long molecular dynamics (MD) simulations with two independent replicas using Desmond, coupled with thermal MM-GBSA calculations for the estimation of the binding free energy values. The structural skeleton of the seven proposed compounds consists of different functional groups and heterocyclic rings that possess anti-cancer activity and exhibit persistent interactions with key residues in binding pockets throughout the MD simulations. STOCK1N-09823 was determined as the most promising hit that promoted the disruption of the interactions R73 (chain A)/N879 and R73 (chain A)/Y884, which are key for SOS1-mediated KRAS activation.

## 1. Introduction

The RAS family is a group of small GTPases that play key roles in signaling pathways controlling cell proliferation, differentiation, and survival [1,2,3]. Among the three RAS genes, the KRAS gene is responsible for encoding KRAS, a protein that plays a role in the RAS/MAPK signaling pathway [4]. RAS gene oncogenic mutations account for about 30% of human cancers such as lung, colon, and pancreatic; KRAS mutations account for 85% of RAS-driven cancer cases [5,6,7]. These mutations are primarily found in residues G12, G13, and Q61, resulting in aberrant signaling by decreasing intrinsic GTPase activity and/or increasing resistance to GTPase-activating protein (GAP)-mediated hydrolysis, which maintains the protein in a constitutively active state [8,9,10]. Notably, the G13D mutation is present in 25% of colorectal cancers driven by KRAS [7]. GAPs maintain the inactive state of RAS by increasing its GTPase activity, which supports the binding between GDP and KRAS [4]. Like the other RAS proteins, KRAS functions as a binary switch, transitioning between a GTP-bound active state and a GDP-bound inactive state [1,11,12]. The transition between the inactive and active states of RAS is regulated by GAPs, such as p120GAP, neurofibromin, type I neurofibromatosis gene products facilitating GTP hydrolysis, and guanine nucleotide exchange factors (GEFs), such as son of sevenless (SOS) catalyzing the loading of GTP to activate RAS [13,14,15,16,17,18,19].

Due to its smooth surface and strong binding affinity for nucleotides, RAS has often been regarded as a difficult or “undruggable” target for drugs, making it challenging for researchers to develop effective small-molecule inhibitors of RAS, including KRAS [20,21]. So far, the FDA has approved two KRASG12C inhibitors, sotorasib (AMG510) and adagrasib (MRTX849), to treat patients with KRASG12C-driven cancers [22]. These covalent inhibitors target the switch-II pocket of KRASG12C by promising target selectivity, prolonged action, and dosage control. However, covalent inhibitors can also increase the risk of off-target effects and toxicity, as well as lead to drug resistance [23]. A recent experimental study [24] focusing on the binding mechanisms and specificity of these drugs reported that adagrasib is strictly KRAS-specific, but not KRASG12C-specific, and sotorasib can bind G12C mutant of NRAS and HRAS in addition to KRAS. On the other hand, clinical trials revealed the acquired resistance to the inhibitors [25,26], leading to their restricted efficacy only in a small subset of KRAS mutant cancers [27].

At that point, targeting the RAS complexes is another possibility, such as the KRAS-SOS1 complex using experimental [22,28,29], and computational techniques [30,31,32,33]. SOS1 has a dominant role in GDP-GTP exchange and due to its unique participation in the negative feedback loop of the KRAS pathway, it has been recognized as a significant tumor target [34]. SOS1 is composed of approximately 1330 residues and contains multiple domains. The catalytic domain of SOS1 called SOScat (residues 551–1050) is necessary for RAS-specific nucleotide exchange activity and consists of a RAS exchanger motif (REM) domain and a CDC25 domain [19]. KRAS-GTP interacts with the allosteric REM site stronger than with the catalytic CDC25 site [35,36]. To effectively activate KRAS at the catalytic CDC25 site, the allosteric site in REM requires an active KRAS recruitment [37]. Hillig et al. discovered SOS1 inhibitor BAY-293 to block KRAS-SOS1 interaction [38]. The same study also showed that in KRASG12C cell models, BAY-293 exhibits synergistic growth-inhibitory activity in combination with the KRASG12C covalent inhibitor ARS-853. On the other hand, Moghadamchargari et al. reported that KRASG13D mutant exhibits strong interaction, particularly with SOScat, and potent small-molecule disruptors of RAS-SOS complexes, including BAY 293, do not dissociate the ternary complex, unless extremely high concentrations were used [28]. The allosteric mechanism behind the strong affinity of the KRASG13D mutant to SOScat was investigated using molecular dynamics simulations, showing a stable conformational state upon binding of KRASG13D-GTP at the allosteric domain [19]. The off-target problem with the SOS1 inhibitors accommodating a quinazoline scaffold found in many EGFR inhibitors, such as BI-3406 [39] and BAY-293 [38] is another important issue, which was later addressed by the phthalazine-based MRTX0902 [40]. SOS1 inhibitors BI 1701963 (NCT04111458) and MRTX0902 (NCT05578092) are in clinical trials.

However, the potent inhibitors developed for SOS1 may not be effective for the ternary complex and/or may lead to adverse side effects. Therefore, exploring new putative binding sites and inhibitors is necessary, especially to disrupt the conformationally stable ternary KRASG13D-SOS1 complex. Allosteric inhibition emerges as the favored, and sometimes sole approach for achieving heightened selectivity or minimizing toxicity [41,42,43,44,45]. In addition, the allosteric inhibitors can modulate protein activity in the presence of endogenous ligands and/or co-factors as they are not competitive binders. SOS1 inhibition might be feasible by targeting the putative allosteric sites. While the allosteric drugs also face drug resistance to mutations and usage of alternative pathways like the orthosteric drugs, the developed SOS1 allosteric inhibitors can be used in combination strategies to simultaneously suppress KRASG12C or with other inhibitors targeting the critical metabolic pathways [46,47].

In this study, we used the elastic network model-based Essential Site Scanning Analysis (ESSA) to identify essential residues whose perturbation alters the frequency distribution of global motions, pointing to putative allosteric sites [48]. ESSA was performed on SOS1 (PDB ID: 5ovi), the binary KRASG12C-SOS1 (PDB ID: 6epm) complex [38], and the ternary KRASG13D-SOS1 (PDB ID: 7kfz) complex [28]. As a second method, the Residue Interaction Network (RIN) was used to reveal hub residues of the ternary complex with a high capacity to receive and send allosteric signaling upon perturbation, such as ligand binding [49,50], which can be successfully predicted with high statistics [51]. Two new putative allosteric sites were identified on the ternary complex according to ESSA and RIN results. Then, to find potential SOS1 inhibitors for RAS-driven cancers, the InterBioScreen database (https://www.ibscreen.com/, accessed on 27 July 2022), consisting of 67,631 natural compounds, was screened for the ligand binding site and the two putative allosteric sites. Natural compounds provide a diversified chemical scaffold that cannot be represented by traditional chemical libraries, opening up new opportunities. Seven potential hit compounds were selected based on the combination of docking score, Prime MM-GBSA binding energy using Glide (Schrödinger Release 2021-4), and binding patterns. We then performed two independent molecular dynamics simulations using Desmond (Schrödinger Release 2020-1) to identify the critical interactions and investigate the dynamics of the complexes of these seven hit compounds at the ligand binding site and putative allosteric sites of the ternary KRASG13D-SOS1 complex. The critical interactions between R73 (KRAS)/N879 (SOS1) and R73/Y884 at the KRAS-SOS1 interface were monitored and noted to be disrupted upon binding of a hit compound at a putative allosteric site, demonstrating the utility of the computational approach followed in this study and the high potential of the hit compounds as KRAS-SOS1 inhibitors.

## 2. Results and Discussion

### 2.1. Prediction of Putative Allosteric Binding Sites on SOS1 and Its Complexes

**Figure 1:**
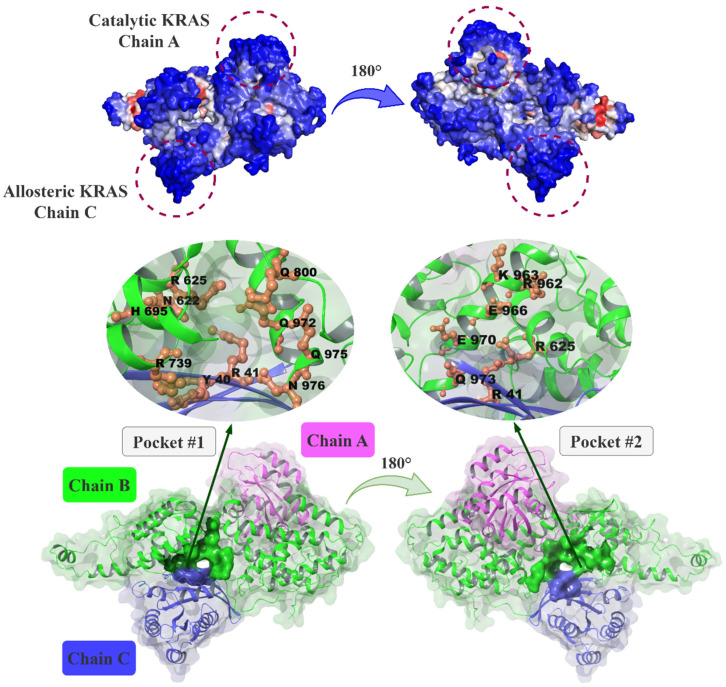
The ternary KRASG13D-SOS1 was viewed from two different perspectives, color-coded by ESSA z-scores from red (highest) to blue (lowest) at the top panel. The putative allosteric pockets P1 and P2 are also shown.

Essential Site Scanning Analysis (ESSA) [48] calculations were performed for SOS1 (PDB ID: 5ovi), the binary KRASG12C-SOS1 complex (PDB ID: 6epm), and the ternary KRASG13D-SOS1 complex (PDB ID: 7kfz) structures to determine the residues essential in their functional dynamics. ESSA calculations provided the z-scores of the residues (Figure S1), which indicate the impact of a perturbation, such as interaction with a ligand on the protein vibrational motions at the low-frequency end of the spectrum. A high z-score underlines the importance of the residue in the protein global dynamics. For each structure, residues with z-scores at the top 25% of the distribution were considered essential [48]. The results are displayed on the tertiary structure in Figure 1 with color codes from high (red) to low (blue) z-scores. The essential residues of the structures with PDB ID: 5ovi, 6epm, and 7kfz are listed in Table S1.

As a second approach, the residue interaction network (RIN) model [49] was applied to detect the residues with high betweenness values in the network, indicating a high capacity to send/receive a perturbation throughout the structure using close residue-residue contacts. The hub residues with high betweenness scores at the top 5% quantile are given in Table S2. To identify the putative allosteric binding sites on SOS1, cavities marked by the mutual residues found both from ESSA and RIN calculations were considered (Table S3).

The calculations suggested hub and/or essential residues both on KRAS and SOS1 structures. ESSA and RIN identified the KRAS switch I (30–40) and II (58–72) residues, and RIN identified the KRAS P-loop including the mutation site G12 as critical that can be evaluated as allosteric binding sites in the KRAS-SOS1 complexes. These regions were also previously proposed by molecular dynamics (MD) simulations [52], solvent mapping technique [53] and high throughput virtual screening coupled with experimental assays [54] for the KRAS structure. It is also worth noting that the conformation of the switch-II residues, such as Y71 and Y64, differs between free and SOS1-bound RAS [55,56]. Here, our computational approach successfully detected the previously suggested allosteric sites of KRAS based on the KRAS-SOS1 complexes.

For the SOS1 structure (PDB ID: 5ovi), SOS1 REM α1 residue R612, SOS1 REM α2 residues F623, F627, L628, Y631, R632 were detected as essential from the ESSA calculations, while Y631 and R632 were also found as hub residues using the RIN model. Furthermore, helical hairpin motif residue S959 is a hub residue according to RIN. Similar regions were found as critical for the globular dynamics of the binary KRASG12C-SOS1 structure (PDB ID: 6epm) according to ESSA. The interdomain residues R625-R632, H700, Y702, R706, Q800-S802, F958, and K960-K963 of SOS1 were identified as essential residues (Table S1) agreeing with the RIN calculations (Table S2). Table S3 lists the mutual residues predicted by two methods. These findings were in high agreement with the previous large-scale MD simulations of the KRAS-SOS1 complex indicating that the KRAS activation is triggered by an allosteric mechanism involving α1, α2, and β1 of the SOS1 REM domain, where R625 and Y631 were highlighted as critical residues [37]. Further supporting our results, a site-directed mutagenesis study demonstrated that mutations of Y64 of RAS and F929 of SOS1, which are predicted as hub residues, decreased the binding affinity of SOS1 to RAS by over 50-fold, whereas the mutation of another hub residue R826 had a smaller effect on the binding and activation of RAS [57]. The same study also showed the role of RAS switch I tyrosine residues Y32 and Y40 found by RIN calculations, in SOS1 mediated guanine nucleotide exchange.

Findings from ESSA and RIN calculations for the ternary KRASG13D-SOS1 structure agreed on the residues Y32, Y40, Y71 (chain A, KRAS), Y631, R688, Y702, Q800, S802, H911, R920, E970, Q975, N976, Y979 (chain B, SOS1) and E37, D38, Y40, R41 (chain C, KRAS.GNP.Mg^2+^) (Table S3). The same regions were also predicted for the SOS1 structure as well as the binary KRASG12C-SOS1 complex underlining these sites as putative allosteric binding sites. Focusing on the druggability score estimations of ESSA, the pocket accommodating solvent-accessible essential/hub residues Y702, S802, Q975, and N976 had a druggability score of 0.049 and a total SASA value of 718.99 Å^2^. In addition, another pocket marked by the solvent-accessible essential/hub residues R920 and E970 had a druggability score of 0.018 with a total SASA of 245.56 Å^2^. The two pockets are located at the interdomain of the KRAS-SOS1 complex and are positioned back-to-back sharing residues R625, Q973 and R41 (chain C). Accordingly, these two pockets called P1 and P2, are promised as druggable sites. The complete list of the residues forming these pockets is given in Table S4 and the pockets are shown in Figure 1.

Supporting our findings, R625, R694, and N976 in P1 were previously proposed as essential residues actively taking a role in the interactions between SOS1 and switch I of KRAS [58]. This implies that these residues have a critical function in directing the allosteric activation of KRAS at the CDC25 location. Moreover, a site directed mutagenesis showed the mutation of L687E/R688A that are suggested as hub residues by RIN calculations, blocked the binding of allosteric RAS at the allosteric site of SOS1 [36]. A previous large-scale MD simulations study [37] reporting the allosteric signaling mechanism in activation cycles of SOS1 systems is in high agreement with our calculations pointing to hub residues Y912, L916, R920, N923, I922, P925 of SOS1 (chain B) and E37 of KRAS (chain C). Another MD simulation study on the ternary complex [19] indicated that residues R694 and W729, I752 and I922 from the SOScat are involved in the allosteric propagation pathway of the ternary complex. The findings from RIN and ESSA calculations also pointed to the high capacity of W729 next to P1, and I922 next to P2 to send/receive an allosteric signal throughout the complex.

### 2.2. Molecular Docking Calculations for the Known Binding Site and Two Putative Allosteric Binding Sites

The docking protocol using Glide XP-docking and Prime MM-GBSA calculations was validated by re-docking the native ligand BQ5 to its binding site in the binary KRASG12C-SOS1 (PDB ID: 6epm) structure. The details of the docking protocol and the results of the re-docking are provided in the Supporting Information (SI), Figure S2. By applying the same procedure, BQ5 was then docked to the corresponding binding site in the ternary KRASG13D-SOS1 complex (PDB ID: 7kfz), yielding an XP-score of −5.61 kcal/mol. The detailed analysis is also given in the SI.

Then, the curated natural compound database (IBS), containing 67,631 compounds at their low energy conformations, was used for the virtual screening targeting the ligand BQ5 binding site and two putative allosteric sites, P1 and P2 pockets, of the ternary KRASG13D-SOS1 complex (PDB ID: 7kfz) in line with the docking protocol. For all binding sites, scores were ranked from the best to the worst, and top compounds with a docking score of 40% higher than the best XP-score were selected. Accordingly, 183 compounds in the ligand binding site; 164 compounds in P1; and 145 compounds in P2 were obtained. These compounds were considered for the Prime MM-GBSA calculations as a further filtration to identify high-affinity compounds at the corresponding sites. The residues, which are kept flexible in the calculations are listed in Table S5. Then, we sorted the Prime MM-GBSA energy values from the best to the worst. We selected the compounds for MD simulations according to their energy values and favorable interactions with the key/essential/hub residues at each site. The followed methodology and the number of compounds after each calculation step are shown in Figure S3.

The Prime MM-GBSA pose of the reference compound BQ5 in the ternary complex indicated that its cyclopenta(c)pyrazole scaffold has a π-π stacked interaction with F890 and with Y884 T-shaped interactions through its phenyl ring (Table S6). The compound BQ5 in the ternary complex was also capable of forming hydrogen bonds with Y884 residue. In addition, π-alkyl and T-shaped interactions were observed with residues L901 and H905, respectively. A previous experimental study showed that the mutations in N879, L901, and H905 led to a decrease in the inhibitory effect of the quinazoline-containing compounds at the binding site on SOS1 [38], indicating the importance of residues L901 and H905 interacting with the BQ5 in the ternary complex Prime MM-GBSA pose.

Fourteen structures for the known binding site of the ternary complex were chosen for MD simulations based on their interactions with the abovementioned key residues, their energies, and scaffolds. All selected compounds have a hydrogen bond interaction with Y884 in their Prime MM-GBSA pose (Table S6). In addition, some of the compounds were in T-stacking contact with Y884 similar to the previous structural studies [38]. The residues F890 and L901, which form the hydrophobic pocket of the ligand binding site, were involved in π-π or π-alkyl type interactions with the aromatic rings of the selected compounds (Table S6). In addition, the key residues D887, H905, and N879 also make favorable interactions with the selected natural compounds.

For P1, a total of 164 compounds were obtained according to the filtering procedure. Among these, twelve hit compounds were selected either based on the non-bonded interactions with the hub residues and the chemical fragments present in their structure or alternatively due to their ability to bind two or more pockets. All compounds have a hydrogen bond interaction with N976 (chain B), and most of them with residues Y40 (chain C), and R41 (chain C) that were predicted by either RIN or ESSA calculations (Tables S1, S2 and S6). Some of the selected structures interact through salt bridges with hub residues D33 and D38 on chain C. The structures that displayed a high Prime MM-GBSA energy value to P1, contain guadinidium, aromatic N-heterocyclic groups, glycosidic linkages and accommodate numerous –OH groups for hydrogen bond interactions both as a donor and an acceptor with most of the residues defining P1 (Table S6).

Pockets P1 and P2 are located in a back-to-back position and share some essential residues as R625, Q973 and R41 (chain C) (Table S4). As for P2, eight compounds came forward, where five of them were also reported for P1. The structures presenting strong hydrogen bond interactions with the residues R625, R962, and R41 (chain C), identified by ESSA analysis, and with residues E966, K595 have better Prime MM-GBSA energies. Even though the compounds have high molecular weights ($\geq 500\;g\cdot {mol}^{-1}$), they were noted to fit P2.

Among the top-ranked compounds 14 for the ligand binding site, 12 for P1, and 8 for P2 were selected based on their structural features and binding patterns (Table S7), and further investigated with the MD simulations to check the stability of the ligand-ternary KRASG13D-SOS1 complex and to analyze the interactions within the binding sites.

### 2.3. Molecular Dynamics Simulations

We first performed 1 μs classical MD simulations for the ternary KRASG13D-SOS1 complex to understand its structural dynamics. Then, the docking poses of 34 hit compounds and BQ5 on the ternary KRASG13D-SOS1 complex were assessed and compared using MD simulations for their stability and conformational changes upon binding of the ligands. For each complex, 2 × 400 ns MD simulations were conducted reaching a total simulation time of 29.0 μs. Overall structural stability and conformational flexibility of the complexes were monitored using the root mean square deviation (RMSD), based on Cα atoms of the ternary complex and the heavy atoms of the ligands. The RMSD of the holo form of KRASG13D-SOS1 ternary structure quickly reached a plateau ~3.5 Å (Figure S4a), agreeing with previous studies [30,58]. The observed increase in RMSD can be attributed to conformational changes in the flexible loops of SOS1, encompassing residues E654-L670, A743-S757, and P1018-N1044, as depicted from the root mean square fluctuations (RMSF) shown in Figure S4b.

**Figure 2:**
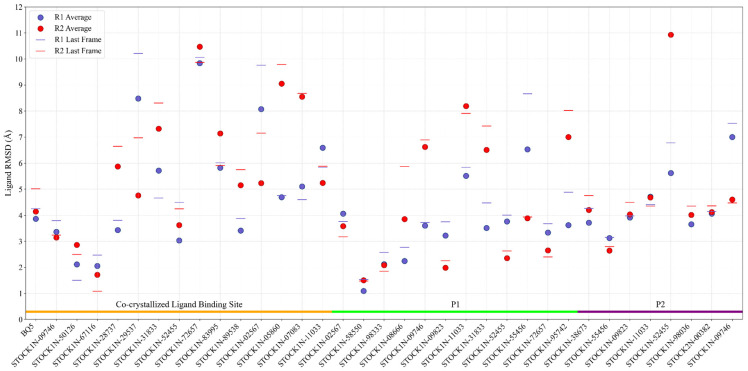
The average (dot) and last frame (dash) root-mean-square deviation (RMSD) of the ligand for each molecular dynamics (MD) simulation of the selected natural compounds and BQ5 are presented. Here, the co-crystalized ligand binding site, allosteric P1, and allosteric P2 are colored in orange, lime, and purple horizontal bars, respectively.

**Figure 3:**
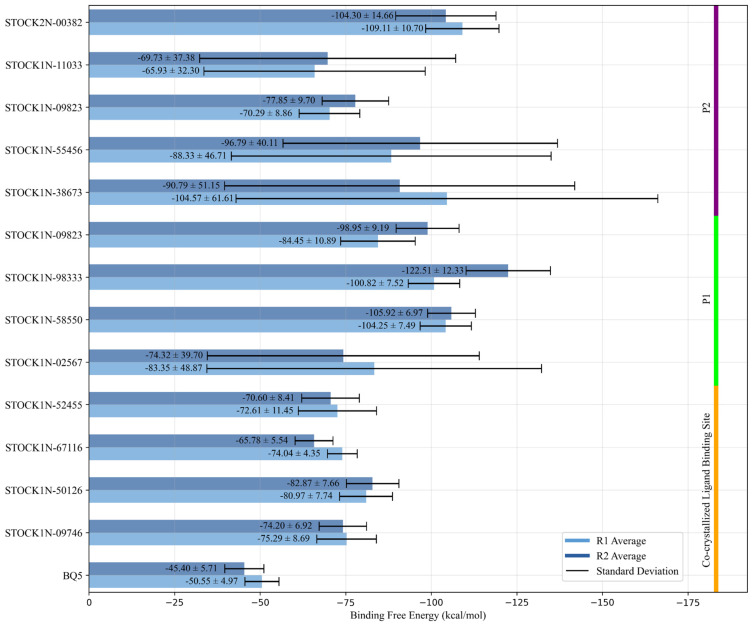
The calculated binding free energies and standard deviations of the selected natural compounds for two molecular dynamics simulation replicas are given in kcal/mol. Same coloring as in Figure 2 is used.

When BQ5 is complexed with the ternary structure, the average RMSD values of the receptor were found to be 2.82 Å and 2.71 Å for each replica. (Figures S5 and S6). The RMSD of BQ5 in the cavity was stable at around 4.0 Å throughout the simulation time where the average RMSD was equal to 3.86 Å in replica 1 (Figure 2 and Figure S5) and in the other replica, it reached to high values of around 8.00 Å between 250 and 350 ns and then decreased to 4.50 Å and the average RMSD was found to be 4.14 Å (Figure 2 and Figure S6). Despite the high RMSD values of the BQ5, particularly for the second replica, the ligand remained in the binding cavity and kept its interactions with the key residues. Indeed, the ligand BQ5 was sandwiched between the delocalized π orbitals of the aromatic side chains of residues Y884 and F890 (Figures S5 and S6). The hydrogen bond interaction was observed most of the simulation time with residues E902 and H905 on chain B in addition to rarely seen water bridges of N879 and D887 residues. Throughout the MD simulations, the persistent hydrogen bond interactions of E902 and H905 with KRASG13D-SOS1 suggests the significance of their roles in ligand stabilization. The binding free energy of BQ5 was estimated as −50.55 ± 4.97 in replica 1 and −45.40 ± 5.71 kcal/mol in replica 2 (Figure 3).

The RMSD results from MD simulations of 34 hit compounds were analyzed for both the receptor and ligand (Figure 2). Compounds with an average RMSD value greater than 5 Å that either escaped from the defined binding site or failed to interact with residues known to play a role in binding within the cavity were excluded from further analysis without calculating their thermal MM-GBSA values. Consequently, based on the MD simulations, 4 compounds for the known binding site, 4 compounds for P1, and 5 compounds for P2 were selected for further analysis (Figure 3).

Molecular docking followed by Prime MM-GBSA calculations pointed out that STOCK1N-09746, STOCK1N-50126, STOCK1N-67116 and STOCK1N-52455 had a better affinity to the known ligand binding site. Indeed, the average ligand RMSDs of those compounds for both MD replicas were less than 4.0 Å indicating the stability of the ligands in the binding site (Figure 2). The average RMSD of the receptors of the complexes ranged between 2.8 Å and 3.7 Å (Figures S7–S14), while the RMSFs of the residues were small, excluding the high peaks of the chain ends and flexible loops, all favoring the stability of the protein complexes with docked compounds. Among the selected natural compounds for the co-crystallized ligand binding site, two of them, STOCK1N-09746 (also known as framycetin) and STOCK1N-50126 can be classified as aminoglycoside antibiotics with a complex structure composed of multiple amino sugars linked to a central aminocyclitol ring. During MD runs, framycetin consistently formed hydrogen bonds with D887 and sometimes with N879 and M878 through water molecules. Additionally, it interacted with Y884 through both hydrogen bonds and hydrophobic interactions, and with R885 through strong hydrogen bond interaction solely. (Figures S7 and S8). These interactions enabled the compound to remain in the binding site throughout the simulations with an average binding free energy of −75.29 ± 8.69 in replica 1 and −74.20 ± 6.92 kcal/mol in replica 2 (Figure 3). Hydrogen bonding interactions were observed between the natural compound STOCK1N-50126, which is structurally similar to antibiotic amikacin, and residues within the binding site, specifically M878, Y884, L886, D887 and E902. Additionally, hydrophobic interactions were observed between this compound and residues Y884 and F890 (Figures S9 and S10). The observed interactions for both compounds are consistent with those found between BQ5 and the KRASG13D-SOS1 ternary complex. Both the calculated binding free energy value of −80.97 ± 7.74 in replica 1 and −82.87 ± 7.66 kcal/mol in replica 2 and its interactions with the key residues including the lower RMSD value make STOCK1N-50126 a better candidate for the co-crystallized ligand binding site (Figure 2 and Figure 3).

Currently reported SOS1 inhibitors include mostly aromatic heterocyclic rings such as quinazolines, and anthraquinones. Heterocyclic compounds have strong interactions with KRAS and NRAS, competing with GDP for the nucleotide-binding site [59]. The heterocyclic nitrogenous compounds known as pyridines and pyrimidines are used to develop anticancer drugs. According to FDA databases, N-containing heterocycles constitute 60% of small molecules, demonstrating the structural significance of these nitrogen-containing heterocyclic compounds in drug discovery [60]. Compound STOCK1N-67116 could be considered as another candidate for the co-crystallized ligand binding site from this perspective. It formed hydrogen bonds with M878, L886, and K898, as well as hydrophobic interactions with Y884 and F890 for over 80% of the simulations. The hydrophobic interactions included π-cation interactions with Y884 and both π-cation and π-π stacking interactions with F890 (Figures S11 and S12).

It should be noted that based on the Prime MM-GBSA binding energies for all three sites, compound STOCK1N-52455 was identified as a common candidate (Table S6). Nevertheless, the results of the conducted MD simulations, particularly those depicted in the last frame, indicate that the molecule in question underwent displacement from the potential allosteric sites, P1 and P2, thereby losing contact with the key residues.

For the potential allosteric P1 located on SOS1, classical MD simulations for the four natural compounds, STOCK1N-02567, STOCK1N-58550, STOCK1N-98333, and STOCK1N-09823 docked to the KRASG13D-SOS1 ternary complex were analyzed in detail. The average RMSD values for the receptor, KRASG13D-SOS1, based on two replicas were calculated around ~3.0 Å indicating the stability of the structures for all selected complexes (Figures S15–S22). The mean ligand RMSD values for the four selected candidates range from 1.1 Å to 4.1 Å (Figure 3), indicating that the compounds have remained within the pocket throughout the simulations. The compound STOCK1N-02567 has a high number of hydrogen bond donor and acceptor groups due to the presence of pyranose rings that facilitate binding to the potential allosteric site. Due to the excess water molecules in this allosteric site, the compound interacted with the receptor mostly by water bridges. However, these types of extensive interaction set a disadvantage of high desolvation energies. The residues keeping the hydrogen bond interaction with the compound throughout the simulation were Q800, Q972, N976, and R41 (chain C) on the SOS1. The calculated average thermal binding energies for replica 1 and replica 2 were −83.35 ± 48.87 kcal/mol and −74.32 ± 39.70 kcal/mol, respectively, even though STOCK1N-02567 remained in the pocket. The high standard deviation in the binding energies is due to the high rotational degree of freedom. This makes it less likely to be a hit compound. Although STOCK1N-58550 is a compound with a lower molecular weight, its calculated binding free energy (−104.25 ± 7.49 kcal/mol for R1 and −105.92 ± 6.97 kcal/mol for R2) is better than STOCK1N-02567 and it has the lowest ligand RMSD value among the four hits for P1. The presence of heterocyclic aromatic rings in the skeleton of the STOCK1N-58550 makes hydrophobic interactions more dominant (Figures S17 and S18). The hydrogen bond interactions were noted especially with N976 of SOS1 and S39 of KRAS (chain C) through nitrogen atoms of amide linkage and carboline ring respectively, where the interactions with the hub residue N976 were conserved in the replicas approximately 90% of the simulation time. The presence of two methoxy groups and the γ-carboline ring in the structure, along with the established importance of these functional groups in the antimetastatic activity of anticancer compounds and binding ability to KRAS, respectively, distinguishes the compound from the other hits studied [61,62]. Moreover, the potential of pyridine rings to serve as promising scaffolds for a variety of anticancer drugs further distinguishes this compound [63]. Freitas et al. (2014) synthesized numerous novel 8-hydroxyquinoline analogs and tested their antiproliferative effectiveness against several cancer cell lines. They also investigated the electronic and steric effects of halogen substituents like fluorine, chlorine, bromine, and iodine linked to aromatic rings where they found that the chlorinated one had the strongest antiproliferative impact [64]. The presence of aforementioned functional groups and nitrogen-containing heterocyclic structure suggests that compound STOCK1N-58550 can be considered as a potential candidate. The compound with the best binding free energy, STOCK1N-98333 (Figure 3), exhibits strong hydrogen bonding and hydrophobic interactions due to its hydrogen bond acceptor and donor groups and aromatic rings. These interactions effectively keep the compound within the active site. Interactions with KRAS (chain C) residues mostly involve hydrogen bonds via water bridges, whereas residues Q972, Q975, and R976 in P1 interact directly with the ligand via hydrogen bonds, especially in the first replica (Figures S19 and S20).

The last structure analyzed for P1, STOCK1N-09823, is also one of the selected compounds for P2 where allosteric pocket P1 shares common residues with its neighbor P2 (Figure 1). The compound, which contains two pyranose rings and a fused aromatic ring, exhibits both high hydrogen bonding capacity and strong π-π interactions with hydrophobic residues. The MD simulations conducted for both regions revealed a persistent interaction with R41, identified as the hub residue for both pockets. Moreover, interactions were observed with residues H699, Q972, Q975, N976, Q25 (C chain), and D38 (C chain) in the case of P1 and with residues D620 and E966 in the case of P2, and the favorable binding energies in both regions point to further investigation of this structure (Figures S21–S24).

Among the structures selected for the P2 pocket, a higher standard deviation of the binding free energies was observed for compounds STOCK1N-55456, STOCK1N-38673, and STOCK1N-11033 (Figure 3). Although all of the compounds interact with the hub residue R41, they appear to interact to a lesser extent with the other residues of the pocket, except for STOCK1N-55456 (Figures S23–S32). These large compounds with many degrees of freedom may have various conformational changes while trying to fit into the binding site. It is possible that peripheral water molecules may occupy critical locations within the binding site, thereby impeding the ligand’s ability to fully engage with the binding site of the target protein. This may result in significant deviations in the calculated thermal MM-GBSA binding energies [65]. The indole derivative, STOCK2N-00382 with hydrogen bond donor amine groups forms hydrogen bonds with D620, E966, E970 and R41 during the MD simulations, while it has hydrophobic interactions through its aromatic ring with the hub residue R41 (Figures S30 and S31) which lead to a better binding energy (−109.11 ± 10.70 for R1 and −104.30 ± 14.66 for R2) than the other pocket P2 compounds. In fact, indole-derived compounds targeting SOS1-mediated RAS signaling present a promising avenue for investigating RAS modulation in cancer therapy [66].

Then, the RMSFs were analyzed for the dynamic behavior and mobilities of the residues. Higher fluctuations > 3.0 Å were noted especially for flexible loops of SOS1 for the holo ternary KRASG13D-SOS1 structure (Figure S4). The fluctuations concentrated around loop residues, 121–126 of chain A (KRAS), 657–670, 766–773, 1020–1024 of chain B (SOS1). The RMSF plots of α-carbon atoms of residues in the BQ5-bound KRAS-SOS1 complex revealed a number of alterations (Figures S5 and S6). The overall reduction in RMSF fluctuations of the BQ5-bound complex indicated a diminished flexibility, particularly for the residues 766–773 of SOS1. Two selected compounds of the co-crystallized ligand binding site, STOCK1N-50126 and STOCKN1-67116, showed a decrease in RMSF values for the same residue range in harmony with the BQ5 bound state (Figures S9–S12). In addition, they exhibited an increase in fluctuations in the residues 658–671 in the REM domain of SOS1, ranging from 654 to 676, and these have been identified as exhibiting disordered behavior based on experimental studies [19]. In this study, an increase in fluctuations in the residues 658–671 was also observed as a result of the binding of the hit compounds STOCK1N-50126 and STOCKN1-67116. The binding of the three selected compounds, STOCK1N-58550 (Figures S17 and S18), STOCK1N-98333 (Figures S19 and S20), and STOCK1N-09823 (Figures S21 and S22), to the allosteric pocket P1 results in a comparable impact on the RMSF values of the KRAS-SOS1 tertiary complex. Upon binding of these compounds, fluctuations in switch I residues (28–32) of catalytic KRAS were suppressed, while those in residues 654 to 668 of SOS1 were increased. The effect on the RMSF values of the natural compounds STOCK1N-09823 (Figures S23 and S24) and STOCK2N-00382 (Figures S31 and S32) when bound to the allosteric pocket 2 is highly similar to the changes observed when the BQ5 structure is bound to its binding site. The notable changes in the mobilities of the residue fluctuations at functional domains distant from P1 and P2 further suggest these sites as allosteric.

The natural hit compound STOCK1N-09823, mutual in P1 and P2, contains flavonoid scaffold, also known as luteolin. Flavonoids show promise as anti-cancer agents, particularly in treating tumors. Jiang et al. (2021) showed that luteolin and apigenin suppress PD-L1 in KRAS-mutant NSCLC cells. The study investigated the anti-tumor effects of flavonoids using in vivo and in vitro models. Findings revealed that luteolin and apigenin effectively inhibited lung cancer cell proliferation, induced apoptosis, and enhanced immune responses by reducing PD-L1 expression in KRAS-mutant NSCLC cells [67,68,69]. Previous studies have highlighted the importance of disrupting the interactions of R73/N879 and R73/Y884. As the inhibitor binds to the pocket, it is expected to disrupt the interaction between R73 and N879 for a better inhibitory effect [29]. Our findings indicate that STOCK1N-09823 significantly disrupts the hydrogen bond and π-cation interactions between R73/N879 and R73/Y884, respectively in the SOS1 ligand binding site when bound to the allosteric pockets particularly P1 (Figure S33). During the MD simulations, the distance between these two residues was observed to increase significantly. In addition to the luteolin scaffold, which has been shown to have a strong inhibitory effect on KRAS-mutant lung cancers, it is of considerable importance to note that STOCK1N-09823, with an allosteric effect, disrupts the interactions between residues R73(chain A)/N879 and R73(chain A)/Y884 within the SOS1 ligand binding domain of the KRAS-SOS1 complex.

STOCK1N-50126 and STOCK1N-67116 for the ligand binding site; STOCK1N-58550, STOCK1N-98333, and STOCK1N-09823 for P1; STOCK2N-00382 and STOCK1N-09823 for P2 were observed to maintain their binding poses and have favorable interactions with the binding site residues. Two-dimensional interaction maps and the binding poses of the hit compounds in the target sites are given in Figure S34. While all proposed compounds have a high capacity to modulate the dynamics of the ternary complex, the binding of STOCK1N-09823 at the putative allosteric site P1 disrupted the critical interactions between KRAS and SOS1, highlighting its high potential as an inhibitor.

In silico analysis of the absorption, distribution, metabolism, excretion (ADME) properties and toxicity of the hit compounds is highly useful in the decision-making process of identifying clinical candidates. Accordingly, we evaluated the pharmacokinetic and physiochemical (ADME) properties of the 7 hit compounds and the SOS1 inhibitors BAY-293, BI-3406 and MTRX0902 (NCT05578092) using the QikProp tool of Maestro (Schrödinger Release 2021-4) [70]. The results are presented in Table S8. The hit compounds STOCK1N-98333, STOCK2N-00382, STOCK1N-67116, and STOCK1N-58550 have pharmacokinetic and physiochemical properties within the desirable limits, except for STOCK1N-58550, with a concern for hERG potassium channels blockage when the values of the known inhibitors are also considered. STOCK1N-50126 and STOCK1N-09823 are suggested to have poor gastrointestinal absorption, no blood–brain barrier penetration, and no oral absorption. It is worth mentioning that there exist various techniques to improve the gastrointestinal absorption [71] and the oral bioavailability of the compounds [72]. The toxicity analysis for the hit compounds is performed with pkCSM web server [73], and the results are given in Table S9. The inhibitors are also included into the analysis for comparison. The hit compounds are predicted to have no AMES toxicity, implying that the drugs are not likely to induce mutations in DNA and eventually lead to cancer, except for STOCK1N-09823, similar to inhibitor BAY-293. The maximum tolerated doses of the hit compounds are comparable to those of the inhibitors. The acute oral rat toxicity (LD_50_) is similar for the compounds and are between 2.39–3.07 mol/kg, whereas the chronic oral rat toxicity (LOALEL) values display a wide range from 0.91 to 7.26 log(mg/kg_bw/day). Hepatoxicity is suggested for STOCK1N-98333, STOCK1N-67116, and STOCK1N-58550, similar to the inhibitors. The hit compounds do not show skin sensitization traits. The findings suggest that the hit compounds have a high potential to be evaluated in clinical trials.

## 3. Materials and Methods

### 3.1. Essential Site Scanning Analysis

Essential Site Scanning Analysis (ESSA) is based on the elastic network model, and is employed to identify essential residues with a high capacity to control the overall dynamics of the protein [48]. In this method, each residue undergoes serial perturbations through the insertion of new nodes at the heavy atoms of its side chains, which replicate the crowding and constraints in the local environment such as due to a ligand binding. Essentiality is measured as the mean shift in the frequency of the softest vibrational modes calculated as

(1) $${∆\lambda }_{k}^{\left(i\right)}\left(\%\right)=\frac{\left(\lambda_{k}^{\left(i\right)}-\lambda_{k}\right)}{\lambda_{k}}\times 100$$

Here, ${∆\lambda }_{k}^{\left(i\right)}\left(\%\right)$ is the percent shift in the eigenvalue $\lambda_{k}^{\left(i\right)}$ of the global mode *k* in response to the crowding near residue *i*. $\lambda_{k}$ is the *k*th eigenvalue obtained for the unperturbed/reference structure. For the robustness of the results, ten slowest modes that usually explain the majority of the overall dynamics are considered. Then, a z-score for each residue is calculated as

(2) $$z_{i}=\frac{\langle {∆\lambda }_{1-10}^{\left(i\right)}\rangle -µ}{\sigma }$$

for the quantification of ligand binding effect to the residue *i*. Here, µ and σ respectively denote the mean and the standard deviation of ⟨${∆\lambda }_{1-10}^{\left(i\right)}$⟩ over all residues.

The residues were sorted based on their z-scores and those lying in the top 25% quartile were considered. After determining the pockets of the structure using Fpocket with its default parameters, an ESSA score is assigned to each pocket, representing the median or maximum of the z-scores of the residues forming that pocket. Then the pockets ranked based on these scores.

Residues that participate in orthosteric and allosteric sites, as well as those that regulate global hinge bending movements, were shown as essential [48]. The binding pockets of the ternary KRASG13D-SOS1 complex were assessed using ESSA and Fpocket [74], as implemented in ProDy 2.0 with default parameters [75]. In all calculations, heteroatoms and water molecules were removed from the crystal structures before calculations.

### 3.2. Residue Interaction Network Model

The residue interaction network (RIN) model is based on the contact topology of the protein structure and describes a protein complex as a network of nodes interconnected by edges. The weight of an edge is calculated using the inverse of the local interaction strength between two residues as

(3) $$a_{ij}=\frac{N_{ij}}{\sqrt{N_{i}\cdot N_{j}}}$$

Here, $N_{i}$ and $N_{j}$ are the number of heavy atoms of residues *i* and *j*, respectively. $N_{ij}$ is the number of heavy atom contacts of *i*, *j* pairs within a cutoff distance of 4.5 Å. The inverse of *a_ij_* is assigned as the weight of the edge between the *i*th and *j*th residues; this model thus relaxes the strong bias towards the covalent bonds [49].

By using the centrality measures of the network, such as betweenness, closeness, and degree, nodes that are critical in the biological activity of the protein can be revealed, as was previously shown for the bacterial ribosome and *S. aureus* pyruvate kinase [49,50]. Especially, a high betweenness score of a node (residue) points to an ability to link distant sites of the network (protein structure), and thus to a high capacity to send/receive information in the form of a perturbation propagated through tertiary interactions.

### 3.3. Dataset Preparation

To determine possible allosteric sites, three *H. sapiens* structures from the protein data bank (https://www.rcsb.org/) [76] were studied. These are SOS1 in complex with small molecule inhibitor BAY-293 (PDB ID: 5ovi [38]), SOS1 in complex with KRASG12C and the co-crystalized fragment at the SOS1 binding site, (1-phenyl-5,6-dihydro-4~{H}-cyclopenta[c]pyrazol-3-yl)methanamine (BQ5 in PDB ID: 6epm [38]), and the ternary KRASG13D-SOS1 complex (PDB ID: 7kfz [28]). The KRASG13D-SOS1 structure, called as ternary complex, is composed of three chains: Chain A (KRAS), Chain B (SOS1), and Chain C (KRAS) (Figure 1). All structures were employed in the determination of putative allosteric sites of SOS1 using RIN and ESSA methods, while the ternary complex was further investigated for molecular docking calculations followed by molecular dynamics simulations for suggesting hit compounds.

Natural compounds from the InterBioScreen (IBS) were used for virtual screening, including a total of 67,631 compounds. Pan-Assay Interference Compounds (PAINS) were applied to the natural compounds [77]. The library was prepared with the LigPrep (Schrödinger Release 2021-4: LigPrep, Schrödinger, LLC, New York, NY, USA, 2021) of Maestro (Schrödinger Release 2021-4: Maestro, Schrödinger, LLC, New York, NY, USA, 2021) using the OPLS_2005 force field [78] at pH 7.4 ± 1.0 with ionization states generated by the Epik module (Schrödinger Release 2021-4: Protein Preparation Wizard. Epik, Schrödinger, LLC, New York, NY, USA, 2021). A total of 67,631 3-dimensional structures were obtained by generating up to 32 stereoisomers for each ligand. Then, the conformers with the lowest energy were selected for docking simulations.

### 3.4. Structure-Based Virtual Screening with Glide

The crystal structures of KRASG12C-SOS1 with co-crystallized fragment BQ5 and the ternary KRASG13D-SOS1 were retrieved from the Protein Data Bank (PDB ID: 6epm and PDB ID: 7kfz, respectively). Molecular docking has been employed to screen the natural product library for its potential to bind to the ternary structure and inhibit its activity. For the validation of the docking protocol, the structure BQ5 was re-docked to its target site in the KRASG12C-SOS1 structure and was docked to the corresponding site at the ternary KRASG13D-SOS1 complex (see the validation protocol details in the Supporting Information for the KRASG12C-SOS1 complex). This computational approach was previously employed by our group to identify potential candidates for the main protease of SARS-CoV-2 and *E. coli* ribosome [79,80].

The ternary KRASG13D-SOS1 complex was prepared using Schrödinger’s Protein Preparation Wizard module for molecular docking calculations. Missing side chains and missing loops were completed with the Prime module. The nucleotide GNP and cofactor Mg^2+^ at the nucleotide-binding pocket were retained. Default settings of the Epik module were kept for a pH of 7.4 ± 1 to determine the protonation states. This was followed by energy minimization to relax the structure. Three different sites, the ligand binding site and two putative allosteric pockets of SOS1 (P1 and P2), were subjected to molecular docking calculations. The OPLS_2005 force field [78] was used for docking calculations. The receptor grid generation module was utilized for the grid box generation. An outer grid box of size 20 Å × 20 Å × 20 Å was chosen. The grid box was centered at x: 107.89 Å, y: 99.51 Å, z: 73.77 Å corresponding to the co-crystallized ligand binding site on ternary KRASG13D-SOS1 structure (PDB ID: 7kfz). The critical residues of P1 and P2 determined from ESSA and RIN calculations were chosen for the outer grid box. Accordingly, the grid box for P1 was centered at x: 86.9 Å, y: 112.26 Å, z: 111.73 Å, while the grid box for P2 was centered at x: 99.25 Å, y: 103.54 Å, z: 113.4 Å. In addition, the inner box size was chosen 10 Å for the generated grid boxes. All molecular docking calculations were carried out using the Glide extra precision (XP) docking with the IBS library comprising 67,631 compounds. The XP docking scores were ranked from best to worst, and the compounds with a score higher than 40% of the top score were considered for Prime MM-GBSA calculations.

### 3.5. Prime MM-GBSA Calculations in Glide

Prime MM-GBSA (molecular mechanics with generalized born surface area) calculations were performed to rank and rescore the XP-docking results of the compounds using the following equation,

(4) $${∆G}_{Bind,\;Prime}=E_{complex}-(E_{protein}+E_{ligand})$$

where, $G_{Bind,\;Prime}$ is the Prime MM-GBSA value for the ligand-protein complex, *E* is the energy of the minimized complex, protein and ligand. Each *E* term includes the $E_{electrostatic}$ ($E_{Coulomb}$ + $H_{bond}$ + $E_{GB}$) and $E_{vDW}$ ($E_{vDW}$ + $E_{\pi \text{-}\pi }$ + $E_{self\text{-}contact}$) terms.

The solvation model, VSGB (Variable-Dielectric Generalized Born), was employed with the OPLS_2005 force field [78] to calculate the ligand binding energy in the Prime MM-GBSA methodology. The residues having both high z-scores in the ESSA analysis and favorable interactions with the docked compounds in the XP-docking poses were selected as flexible in the calculations. Accordingly, N879, Y884, D887, F890, E902, and H905 (chain S) residues were chosen as flexible for the ligand binding site on the ternary complex KRASG13D-SOS1. The P1 residues R625, R694, H695, Y702, Q972, Q973, Q975, P801, S802, N976 and N38, S39, Y40, R41 (chain C) and the P2 residues I600, N622, R625, T626, R920, F958, R962, K963, E966, E970, Q973, and R41, L42, D54 (chain C) were selected as flexible residues. We employed Prime MM-GBSA calculations on 183 compounds for the ligand binding site, 164 compounds for P1, and 145 compounds for P2 to filter and analyze the receptor-ligand binding modes generated by the XP-docking calculations. The compounds with better binding energies than the co-crystallized compound and favorable interactions with essential residues have been selected for molecular dynamics simulations to observe the stability of the complex and the dynamics of predicted binding conformations over time. All the interactions were analyzed and displayed using Discovery Studio Visualizer [81] otherwise stated.

### 3.6. Molecular Dynamics Simulations

The molecular dynamics (MD) simulations of selected ligand-ternary protein complexes were performed using the Desmond simulation package of Schrödinger (Schrödinger Release 2020-1, D. E. Shaw Research, New York, NY, USA) [82]. The initial structures were obtained from Prime MM-GBSA poses. In the first step, the ternary KRASG13D-SOS1 complex with the docked ligand was solvated using the TIP3P water model [83] with an orthorhombic box through a 10 Å padding. All simulation systems were neutralized by adding Na^+^ ions. Then, 0.15 M NaCl ionic strength was applied to mimic the physiological conditions. The solvated system was minimized with a 50 kcal/mol restraint, including ligands.

The standard pre-equilibration protocol of Desmond consists of five steps. It starts with small time steps, at 10 K, 100 ps Brownian Dynamics in the NVT (constant number of particles, constant volume, and constant temperature) ensemble. MD is then applied with constraints on the solute heavy atoms at 10 K, 12 ps in the NVT ensemble. MD follows this step with constraints on the solute heavy atoms at 10 K, 12 ps in the NPT ensemble. Then, MD is performed at the target temperature of 300 K for 12 ps in the NPT ensemble with restraints on the solutes except hydrogens. In the last step, MD is performed without restraints at the target temperature of 300 K for 24 ps in the NPT ensemble. The standard protocol was revised by increasing the length of the last step of the equilibration protocol to 100 ps to further relax the inserted loops at 300 K and a pressure of 1.013 bar. In the production step, the solvated protein-ligand complex system of ~90,000 atoms was subjected to 400 ns long MD simulations with two independent replicas under the OPLS_2005 force field [78]. NPT ensemble was applied along with Nosé–Hoover thermostat [84] and Martyna-Tobias-Klein barostat [85]. The cutoff radius for the Coulombic interactions was taken as 10 Å. The trajectory was recorded every 400.0 ps.

The stability of the simulated systems was monitored by the root mean square deviation (RMSD) of the Cα atoms of the protein and the heavy atoms of the ligand, using the initial frame of the production run as the reference structure. The root mean square fluctuation (RMSF) values of the amino acids and the type of non-bonded interactions between the ligand and the protein, were also calculated. The thermal mmgbsa.py script in the Prime MM-GBSA module of Maestro was employed to estimate the binding free energies (ΔG_bind_) for the selected compounds for each replica. In these calculations, 200 frames out of 1000 frames were considered. The binding energy was estimated according to Equations (5) and (6):

(5) $${∆G}_{bind}={∆E}_{MM}+{∆G}_{solv}+{∆G}_{SA}$$

Here, ${∆E}_{MM}$ is the difference in minimized energies between complex, ligand, and protein-energy as following:

(6) $${∆E}_{MM}=E_{complex}-E_{ligand}-E_{receptor}$$

Here, ${∆G}_{solv}$ denotes the difference in GBSA solvation energy of the complex and the sum of ligand and protein solvation energies. At the same time, ${∆G}_{SA}$ is the differences in surface area energy of the complex and the sum of protein and ligand [86,87]. The entropic contribution to ligand-binding affinity is neglected.

### 3.7. Prediction of the Pharmacokinetic Properties and Toxicity

The analysis of the pharmacokinetic properties is carried out with QikProp tool of Maestro [70] to predict their absorption, distribution, metabolism, and excretion (ADME) properties. The findings are used to predict the kinetic behavior of the compounds to understand their safety and efficacy under appropriate dose regimens that are critical in clinical trials. The QikProp tool calculates a set of parameters to decide on the ADME properties of the compound, namely its molecular weight, the π and weakly polar components of its solvent accessible surface area, the number of hydrogen donors and acceptors, its lipophilicity, its solubility, its blood–brain barrier penetration, its uptake from the gastrointestinal system, and its oral absorption capacity.

The toxicity analysis of the hit compounds is done with the pkCSM web server [73] to reveal their potential toxic effects. AMES toxicity, oral rat acute and chronic toxicities, hepatotoxicity, maximum tolerated dose of the compounds, and skin sensitization are evaluated.

## 4. Conclusions

RAS oncoproteins have been a well-researched family of target proteins and can be targeted by orthosteric and allosteric drugs, as well as their combinations. Targeting KRAS-driven cancers may be achieved by interfering with the activation of KRAS by SOS1. Herein, we report a comprehensive study to identify potential inhibitors for the ternary KRASG13D-SOS1 complex which plays a pivotal role in the nucleotide exchange process, a crucial step in RAS signaling. We first employed the Essential Site Scanning Analysis (ESSA) and Residue Interaction Network (RIN) methods to determine key residues and putative allosteric sites in SOS1 and KRAS-SOS1 complexes (PDB IDs: 5ovi, 6epm, and 7kfz). ESSA and RIN predicted two potential allosteric pockets of the KRASG13D-SOS1 ternary complex structure. The allosteric pocket P1 comprises the interdomain interface between REM and CDC25 of SOS1, including residues Y702, S802 of SOS1, and the KRAS (chain C) switch-I residues D38, Y40, R41. The allosteric P2 neighbors P1, and shares some common residues with P1, including N622, R625, G969, Q973 of SOS1 and R41 of KRAS (chain C). The findings from RIN and ESSA calculations also pointed to the high capacity of R694, W729, and I922 to send/receive an allosteric signal throughout the complex. Then, to discover new inhibitors, 67,631 natural compounds from the InterBioScreen database were screened with extra precision docking (Glide XP) followed by MM-GBSA calculations as implemented in Prime module of Maestro. Among the top-ranked compounds, 34 promising compounds were selected based on docking scores, binding energy, and patterns. Molecular dynamics (MD) were conducted in conjunction with thermal MM-GBSA calculations for these potential hits to compute the relative binding free energy, thereby providing structural and energetic insights into the ability of the hit compounds to bind to the target sites. MM-GBSA approach based on the MD simulations usually provides a good estimation of the binding free energy in the rankings of hit compounds while the estimation can be further improved such as by the implementation of AI [88].

As a result of the extensive analyses performed for MD trajectories of selected natural hit compounds and comparisons made with previous studies, two structures were proposed for the co-crystallized binding site (STOCK1N-50126, STOCK1N-67116) and three and two structures for the potential allosteric sites P1 (STOCK1N-58550, STOCK1N-98333, STOCK1N-09823) and P2 (STOCK2N-00382, STOCK1N-09823), respectively. Throughout the MD simulations, the selected compounds exhibited persistent interactions with the key residues within their binding pockets. It should be noted that the structural scaffold of these compounds is comprised of distinctive functional groups and heterocyclic rings, which have been demonstrated to exhibit anti-cancer activity. Among these compounds, STOCK1N-09823, identified as a hit for both potential allosteric sites, is proposed as the strongest candidate because it contains a flavonoid scaffold, also known as luteolin, and disrupts the interactions between residues R73(chain A)/N879 and R73(chain A)/Y884 within the SOS1 ligand binding domain of the KRAS-SOS1 complex. In addition, in silico analysis of the pharmacokinetic, physicochemical properties and toxicity of the hit compounds showed the high potential of the compounds as clinical candidates.

The strategy followed for KRAS.SOS1 ternary complex can be extended to other RAS proteins involved in tumor progression, such as targeting the HRAS.SOS1 ternary complex with known 3-dimensional structure [36]. While KRAS has a high sequence similarity to other RAS proteins, their hypervariable regions are different, they have different expression levels, membrane attachment status, clustering behavior, and subcellular localization in different cell types [89]. These differences can also affect the potential off-target effects of the proposed hit compounds. Therefore, to reveal the inhibitory effects of the proposed natural compounds on the KRAS.SOS1 complex and the existence of the off-targets, further in vitro/in vivo studies should be carried out.

## Data Availability

All data generated or analyzed during this study are included in the article and the Supplementary Materials.

## Supplementary Materials

The following supporting information can be downloaded at: https://www.mdpi.com/article/10.3390/ijms26073293/s1.
