# Supplementary material for: In Silico Identification of Putative Allosteric Pockets and Inhibitors for the KRASG13D-SOS1 Complex in Cancer Therapy
Source: Int J Mol Sci. 2025 Apr 2;26(7):3293. doi: 10.3390/ijms26073293 (PMC11989364; doi:10.3390/ijms26073293)
Supplement: Supplementary file 1 [file ijms-26-03293-s001.zip › ijms-3515301-supplementary.pdf]

SUPPLEMENTARY MATERIALS

In Silico Identification of Putative Allosteric Pockets and Inhibitors for the KRASG13D-SOS1 Complex in Cancer Therapy

Zehra Sarica [1], Ozge Kurkcuoglu [2,*] and Fethiye Aylin Sungur [1,*]

[1] Computational Science and Engineering Division, Informatics Institute, Istanbul Technical University, Istanbul 34469, Türkiye; sarica16@itu.edu.tr
[2] Department of Chemical Engineering, Istanbul Technical University, Istanbul 34469, Türkiye
* Correspondence: aylin.sungur@itu.edu.tr (F.A.S.); olevitas@itu.edu.tr (O.K.)

Table S1. The essential residues predicted by ESSA calculations.

PDB ID: 5ovi	PDB ID: 6epm	PDB ID: 7kfz
F577, R612, F623, F627, L628, T629 Y631, R632, F648, E652, P653, E654, P655, S671, E673, L674, K675, F677, R678, Y681, Q683, Q686, W696, H700, F701, Y702, F704, E705, R706, L776, Y796, Q800, R826, H827, W834, F835, F868, L872, Y912, Y915, F929, F930, I932, Y933, F958, R962 Y974, Y979, F991.	Y40, R68, Y71, F156, Y157, M567, R568, L569, H616, M617, R625, T626, F627, L628, T629, T630, Y631, R632, F648, F677, E680, Y681, I682, Q683, Q686, W696, H700, Y702, R706, Q800, P801, S802, W834, H911, F958, K960, R961, R962, K963.	Y4, Y32, Y40, R41, K42, I46, L52, L53, R68, Y71, R102, F156, Y157(Chain A)<br>R568, F577, N622, F623, V624, R625, T626, F627, L628, T629, T630, Y631, R632, E652, P653, E654, P655, D659, S671, E673, L674, K675, F677, R678, E680, Y681, R688, N691, R694, H695, W696, H700, F701, Y702, R706, W729, R739, Q800, P801, S802, W834, F835, H911, R920, F958, K960, R962, K963, V964, E970, Q972, Q973, Q975, N976, Q977, Y979, F1010 (chain B)<br>D33, E37, D38, Y40, R41(Chain C)

Table S2. The residues with high betweenness scores predicted by RIN calculations.

PDB ID: 5ovi	PDB ID: 6epm	PDB ID: 7kfz
Y631, R632, H700, Y702, Y796, R797, A798, V799, Q800, P801, S802, E803, S818, L822, R826, T829, N830, F862, P928, F929, S959, R962, A965, T968, I971 (chain A)	G10, S17, I21, Y32, S39, Y40, Y64, R97, K101, R102 (chain R)<br>G604, T605, Y631, R632, L640, H700, Y702, L788, Y796, V799, Q800, P801, S802, E803, S818, L822, I825, R826, N867, H911, V927, P928, F929, G931, N944, P945, I956, N957, S959, K960, A965, T968 (chain S)	G12, D13, I21, Y32, S39, Y40, T58, Q61, E62, Y71, M72, R73 (chain A)<br><br>G604, T605, Y631, R688, Y702, Q755, S756, S757, P758, P759, V761, L788, Y796, V799, Q800, S802, E803, N820, L821, F862, N867, H911, Y912, L916, R920, I922, N923, P924, P925, C926, V927, P928, F929, G931, N944, P945, I956, N957, E970, Q975, N976, Y979 (chain B)<br><br>S17, Q22, E37, D38, Y40, R41, Q43, V45, I46, D57, R149 (chain C)

Table S3. The mutual residues predicted by ESSA and RIN calculations.

PDB ID: 5ovi	PDB ID: 6epm	PDB ID: 7kfz
Y631, R632, H700, Y702, Y796, Q800, R826, F929.	Y40 (chain R),<br>Y631, R632, H700, Y702, Q800, S802, H911, K960 (chain S).	Y32, Y40, Y71(chain A), Y631, R688, Y702, Q800, S802, H911, R920, E970, Q975, N976, Y979 (chain B) and E37, D38, Y40, R41(chain C)

Table S4. Putative allosteric binding sites.

Pocket #1	Pocket #2
P621, N622, V624, R625, T629, N691, R694, H695, Y702, R739, P801, S802, G969, Q972, Q973, Q975, N976 (Chain B)<br>D38, S39, Y40, R41 (Chain C)	P599, I600, I601, D620, N622, F623, R625, T626, R920, F958, R962, K963, E966, I967, G969, E970, Q973 (Chain B)<br>R41, L52, D54 (Chain C)

Table S5. Flexible residues selected for Prime MMGBSA calculations.

Co-crystallized ligand binding site	Pocket #1	Pocket #2
N879, Y884, D887, F890, E902, and H905	R625, R694, H695, Y702, Q972, Q973, Q975, P801, S802, N976 (chain B) and N38, S39, Y40, R41(chain C)	I600, N622, R625, T626, R920, F958, R962, K963, E966, E970, Q973(chain B), and R41, L42, D54 (chain C)

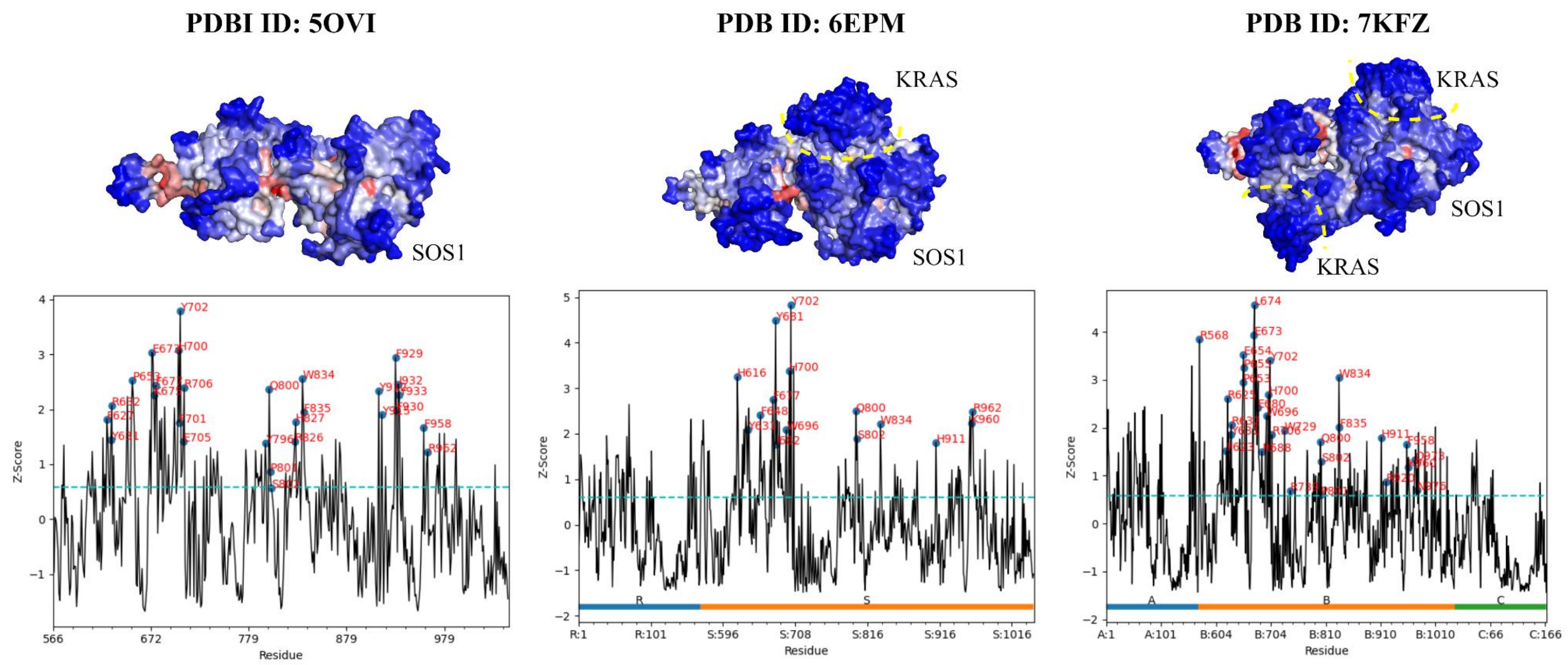

Figure S1. Z-scores of the residues identified from ESSA calculations for SOS1 (PDB ID: 5ovi), KRAS-SOS1 (PDB ID: 6epm) and ternary KRASG13D-SOS1 (PDB ID: 7kfz). The structures at the top panel are color-coded from blue (low Z-score) to red (high Z-score).

1. The Docking Protocol

In this study, we have used the crystal structure of the KRASG12C-SOS1 complex (PDB ID: 6epm) with (1-phenyl-5,6-dihydro-4~{H}-cyclopenta[c]pyrazol-3-yl) methanamine as the co-crystalized ligand. The protein structure was prepared in the Protein Preparation Wizard module in Maestro. Missing side chains and missing loops were completed with the Prime module. Default settings of the Epik module were kept for a pH of 6.5±1 to determine the protonation states. This was followed by energy minimization to relax the structure. The heteroatoms and water molecules were removed before the calculation. Furthermore, the receptor grid generation module was utilized for the grid box generation. An outer grid box of size 20Å × 20Å × 20Å was chosen with the inner box size 10 Å. The grid box was centered at x: 183.41 Å, y: 120.87 Å, z: 279.33 Å. To validate the docking protocol, firstly, the structure BQ5 was extracted and re-docked to its target site in the structure. The re-docked ligand XP pose was superimposed onto the co-crystallized ligand pose using a superposition module of Maestro, and an RMSD of 1.22 Å was obtained. The docking score of the BQ5 in the target site was -8.38 kcal/mol, and the Prime MM-GBSA dG bind score of -68.72 kcal/mol. This alignment confirms that the docking methodology is robustly reliable and can be applied to further virtual screening studies.

The co-crystallized ligand BQ5 of KRASG12C-SOS1 structure interacted with binding site residues Y884 and D887 via hydrogen bond, L901 via π-σ, F890 via π-π stacked and π -alkyl, H905 via π-π T-shaped, V883 and M878 via alkyl. Glide XP docking pose of the structure with the ligand conserved interaction of Y884 via hydrogen bond, F890 via π-π stacked and π -alkyl, H905 via π-π T-shaped, V883 and M878 via alkyl. Also, prime MMGBSA pose of the structure with the ligand conserved interactions of H905 via π-π T-shaped, F890 via π-π stacked and π -alkyl, and Y884 via hydrogen bond. The poses of BQ5 obtained from XP and Prime docking studies are in harmony with the that of the crystal structure.

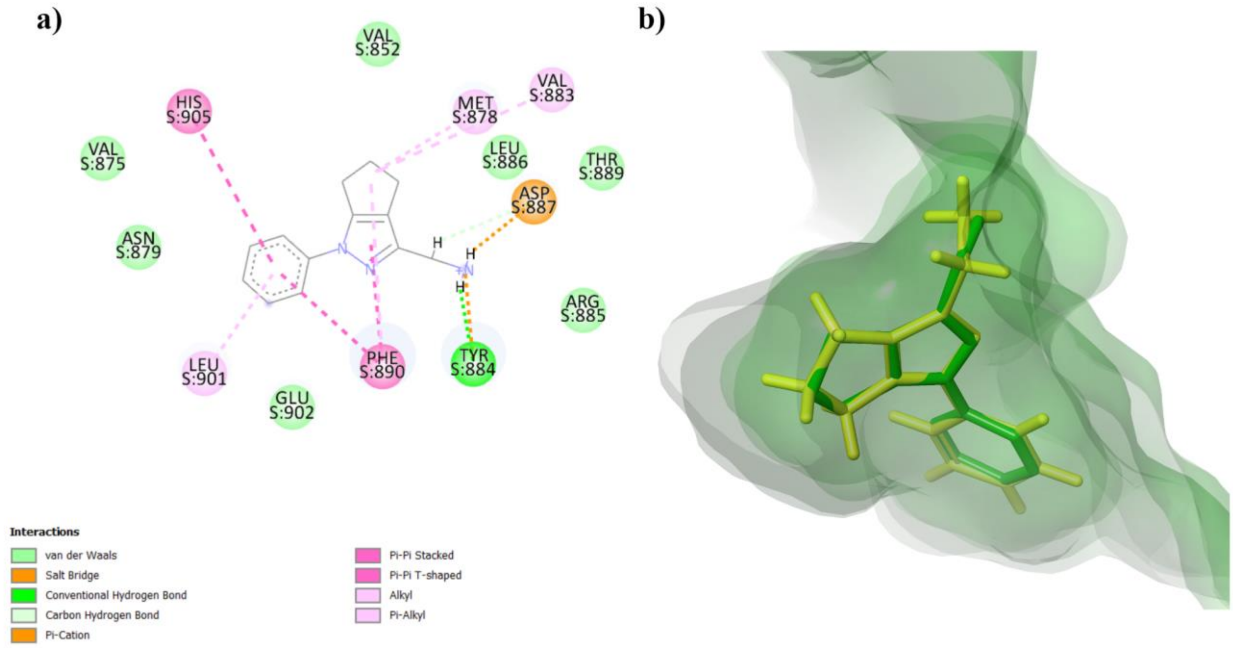

Figure S2. a) The 2D protein-ligand interactions of the KRASG12C-BQ5 complex obtained by Glide XP docking **b)** The superimposed of the co-crystallized ligand BQ5 of KRASG12C-BQ5 complex XP re-docked pose (yellow) and the crystal KRASG12C-BQ5 complex (green) with RMSD of 1.22 Å.

Secondly, the BQ5 from the KRASG12C-SOS1 structure was docked in the site of the ternary KRASG13D-SOS1 (PDB ID: 7kfz) complex corresponding to its position at the KRASG12C-

SOS1 complex. The docking score of the structure BQ5 on its target site of the ternary KRASG13D-SOS1 complex was -5.61 kcal/mol, and the Prime MM-GBSA dG bind score of -34.12 kcal/mol. The protein-ligand interaction 2D diagram of XP pose of the ternary KRASG13D-SOS1 complex was revealed that the ligand interacted with binding site residues Y884 via hydrogen bond and π-cation, F890 via π-π stacked, H905 via π-π T shaped, and L901 via π-alkyl interaction. Considering the ligand-receptor interactions in the crystal structure, most of the interactions are conserved for the XP pose of the ternary KRASG13D-SOS1 complex.

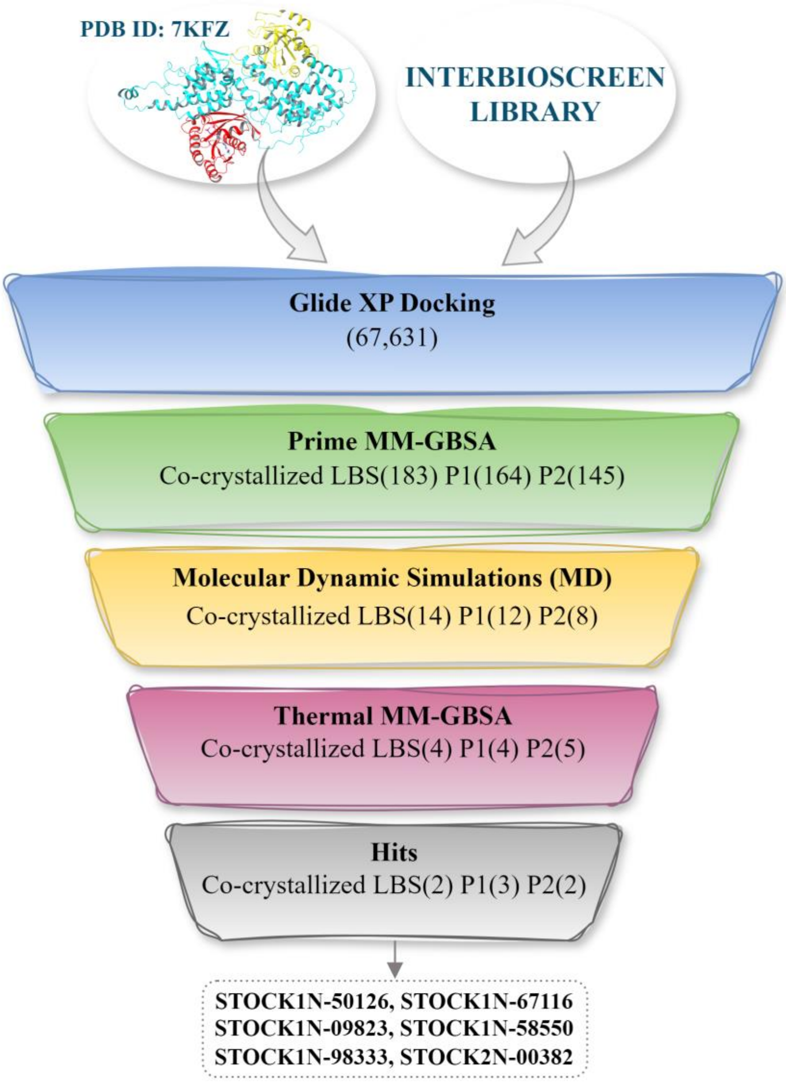

Figure S3. The methodology followed in this study is shown with the number of compounds in parentheses after each calculation step to suggest hit compounds as potential inhibitors.

2. Prime MM-GBSA Calculations

Table S6. Prime MM-GBSA binding pose analysis of the selected compounds.

Site	Compounds	XP GScore (kcal/mol)	Prime MMGBSA ΔG bind (kcal/mol)	Hydrogen Bond	π-Cation	π – π Stacked or T-shaped or π-Alkyl	Salt Bridge; Attractive Charge
Co-crystallized ligand binding site	BQ5	-5.61	-34.12	Y884		Y884, F890, H905, L901	
	STOCK1N-83995	-8.40	-82.39	R73, K104 (chain A), M878, N879, Y884 (chain B)	R73 (chain A)	F890, Y884, L901, H905 (chain B), R73 (chain A)	
	STOCK1N-31833	-9.15	-82.24	Y884, L886, D887, E902, H905 (chain B), D105 (chain A)	F890 (chain B)	Y884, F890 (chain B)	D105 (chain A), E906 (chain B)
	STOCK1N-89538	-8.47	-77.67	M878, Y884, E902, H905 (chain B)	Y884, F890 (chain B)	H905 (chain B)	
	STOCK1N-29537	-9.64	-76.41	V103, K104 (chain A), Y884 (chain B)	Y884 (chain B)	Y884, L901 (chain B)	
	STOCK1N-50126	-9.55	-72.85	M878, Y884, R885, D887, L901 (chain B)	H905 (chain B)		D887, E902 (chain B)
	STOCK1N-07083	-10.98	-72.85	Y884, R885, E902 (chain B)	Y884, F890, K898 (chain B)	H888, R1019 (chain B)	D887 (chain B)
	STOCK1N-28737	-8.09	-72.39	Y884, E902 (chain B)	Y884 (chain B)	F890, Y884, H905 (chain B)	E902 (chain B)
	STOCK1N-67116	-9.06	-71.20	M878, Y884 (chain B)	F890 (chain B)	F890, H905, L901 (chain B)	
	STOCK1N-11033	-8.97	-69.66	Y884, R885, K899, E902, E909 (chain B), D105 (chain A)			
	STOCK1N-02567	-13.21	-66.98	R73, V103, D105 (chain A), Y884, R885, D887,		Y884 (chain B)	

				K898, E902 (chain B)			
	STOCK1N-72657	-8.12	-66.42	Y884, E902 (chain B)	Y884, F890 (chain B)		E902 (chain B)
	STOCK1N-09746	-9.56	-64.68	D105 (chain A), Y884, R885 (chain B)			D887 (chain B)
	STOCK1N-05860	-9.90	-61.47	Y884, D887, E891, N1020 (chain B)	H888 (chain B)		D887 (chain B)
	STOCK1N-52455	-9.29	-56.97	N879, Y884, E902, E906, E909 (chain B)	Y884, H905 (chain B)		E902, E909 (chain B)
**Allosteric pocket P1**	STOCK1N-52455	-14.03	-113.07	E698, Q800, Q972, N976 (chain B), Q25, D33, D38, Y40 (chain C)		H750 (chain B)	D33 (chain C)
	STOCK1N-98333	-10.19	-109.52	N622, R625, Q800, N976 (chain B), R41 (chain C)		H695, H699, H700, H750 (chain B), R41 (chain C)	
	STOCK1N-09746	-11.51	-107.15	R694, E698, H699, R739, Q972, Q975, N976 (chain B), Q25, Y40 (chain C)			E31, D33, D38 (chain C), E698 (chain B)
	STOCK1N-95742	-10.98	-106.93	Q800, G969, Q972, Q975, N976 (chain B), S39, R41 (chain C)			E698 (chain B)
	STOCK1N-58550	-10.17	-101.68	S39 (chain C), H699, N976 (chain B)		Y40 (chain C), H695, H699, H750, R797 (chain B), Y40, R41 (chain C)	
	STOCK1N-08666	-11.40	-100.80	E698, R797, Q800, N976 (chain B), Q25, D33, D38 (chain C)		H699, P801 (chain B)	R739 (chain B), D33, D38 (chain C)

	STOCK1N-31833	-10.24	-93.31	E698, H699, R739, K740, R797, Q800, Q975, N976 (chain B), Q25, Y40 (chain C)		H695, H699 (chain B), H27, Y40 (chain C)	D33 (chain C), E705 (chain B)
	STOCK1N-72657	-10.33	-88.83	S39 (chain C), H699 (chain B)		Y40 (chain C), R41 (chain C)	E698 (chain B), D33, D38 (chain C)
	STOCK1N-09823	-14.40	-85.85	N622, H699, R797, Q800, G969, Q975, N976 (chain B), D38, R41 (chain C)		H699 (chain B), Y40 (chain C), R41 (chain C)	
	STOCK1N-02567	-17.99	-85.52	E698, H699, Y702, E705, R739, K740, R797, Q800, Q975, N976 (chain B), Q25, S39, R41 (chain C)		H695, H699 (chain B)	
	STOCK1N-55456	-12.72	-57.89	E705, R739, K740, N976 (chain B), Q25, H27, R41 (chain C)		H750 (chain B)	
	STOCK1N-11033	-15.19	-52.45	H699, Y702, E705, R739, R797, Q800, Q975, N976 (chain B), E31 (chain C)			
**Allosteric Pocket P2**	STOCK1N-55456	-11.47	-107.97	E589, K595, R625, H699, R962, E966, N976 (chain B), E3, Q25, S39, R41 (chain C)			
	STOCK1N-38673	-11.29	-106.71	E590, M592, K595, N622, R625, R962, K963 (chain B), M1, R41 (chain C), E31, D33 (chain A)			

	STOCK2N-00382	-9.62	-98.48	K595, I598, R625, E966 (chain B), R41 (chain C)	R41 (chain C)	K595, A596, I600, F623, F958, R962 (chain B), L52 (chain C)	
	STOCK1N-11033	-15.97	-91.64	E589, E590, M592, R625, T626, R962, E966, E970, Q973 (chain B), D33, T35 (chain A), E3 (chain C)			
	STOCK1N-09823	-11.52	-82.71	E590, M592, Q593, K595, D620, N622, R962, E966, Q973 (chain B)	R41 (chain C)		
	STOCK1N-52455	-11.04	-69.43	E589, E590, M592, Q593, K595, R962, E966 (chain B), E3 (chain C)			E589, D620 (chain B)
	STOCK1N-98036	-9.56	-82.59	E590, Q593, N622, R920, E966 (chain B)	K595 (chain B)	A596, I600 (chain B)	
	STOCK1N-09746	-11.19	-66.37	E589, M592, K595, N622, T626 (chain B)			D620, E966 (chain B)

Table S7. 2D structures of the top hits at the co-crystallized ligand binding site, the potential allosteric P1, and the potential allosteric P2 of the ternary KRASG13D-SOS1 complex (PDB ID: 7kfz).

	**Compound ID**	**Structure**
**Co-crystallized ligand binding site**	BQ5	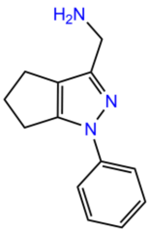
	STOCK1N-09746	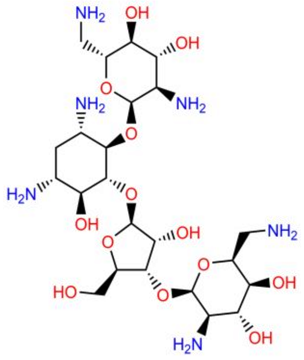
	STOCK1N-50126	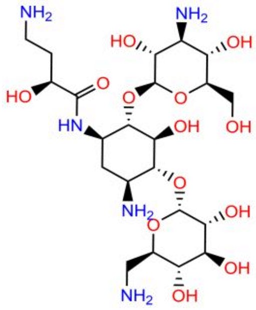
	STOCK1N-67116	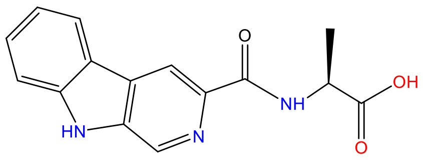
	STOCK1N-52455	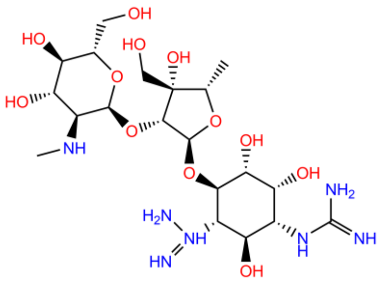

**Allosteric Pocket P1**	STOCK1N-58550	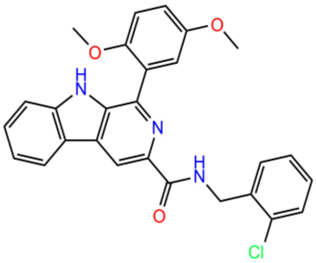
	STOCK1N-98333	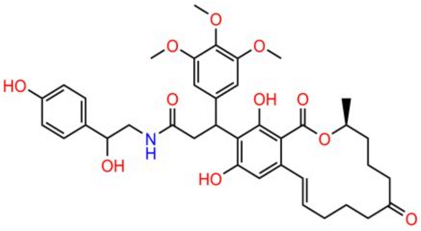
	STOCK1N-02567	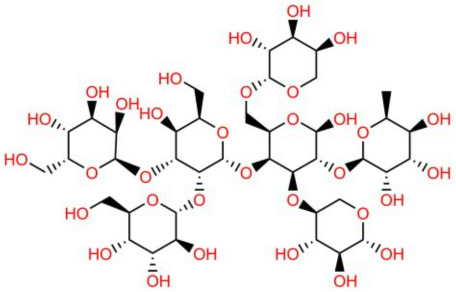
	STOCK1N-09823	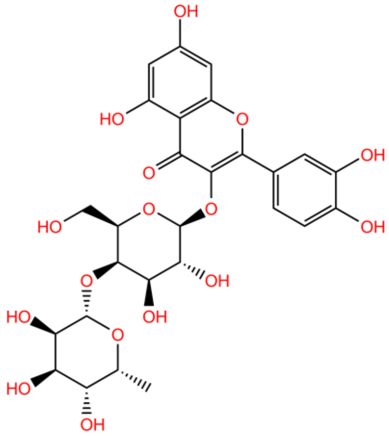
**Allosteric Pocket P2**	STOCK1N-55456	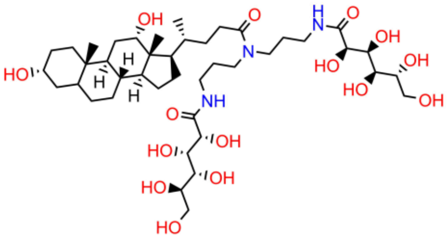

	STOCK1N-38673	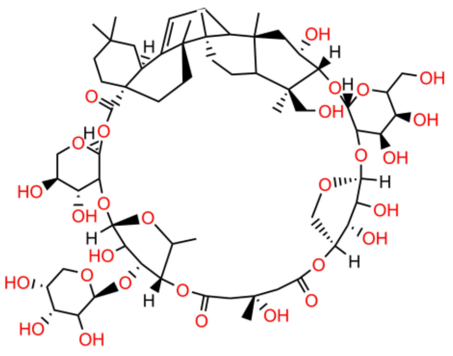
	STOCK1N-09823	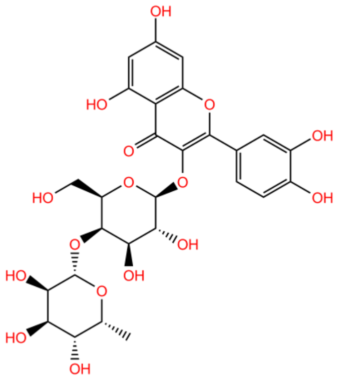
	STOCK1N-11033	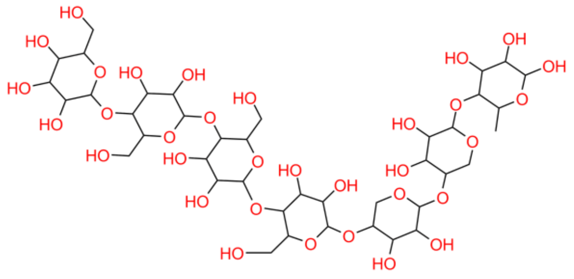
	STOCK2N-00382	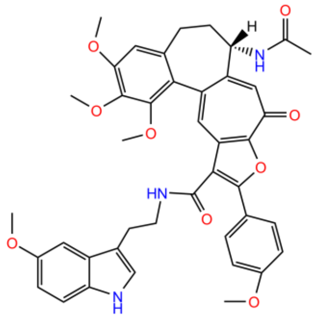

3. Molecular Dynamics Simulations

3.1. The KRASG13D-SOS1 Structure

a)

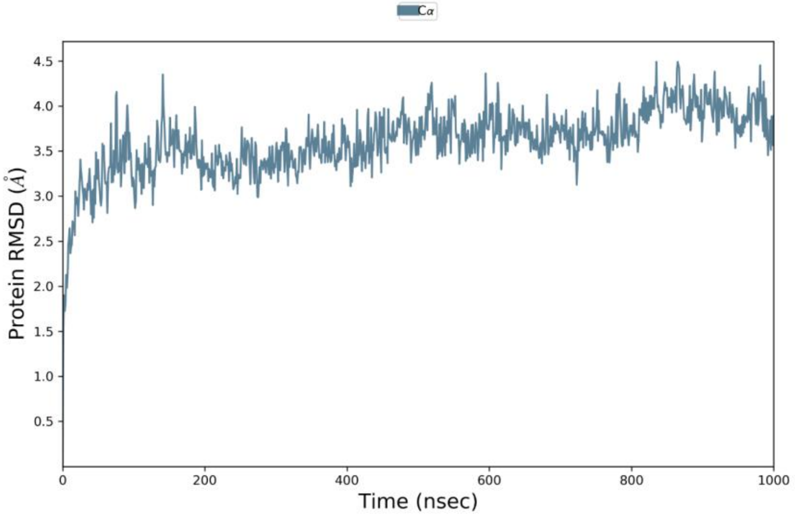

b)

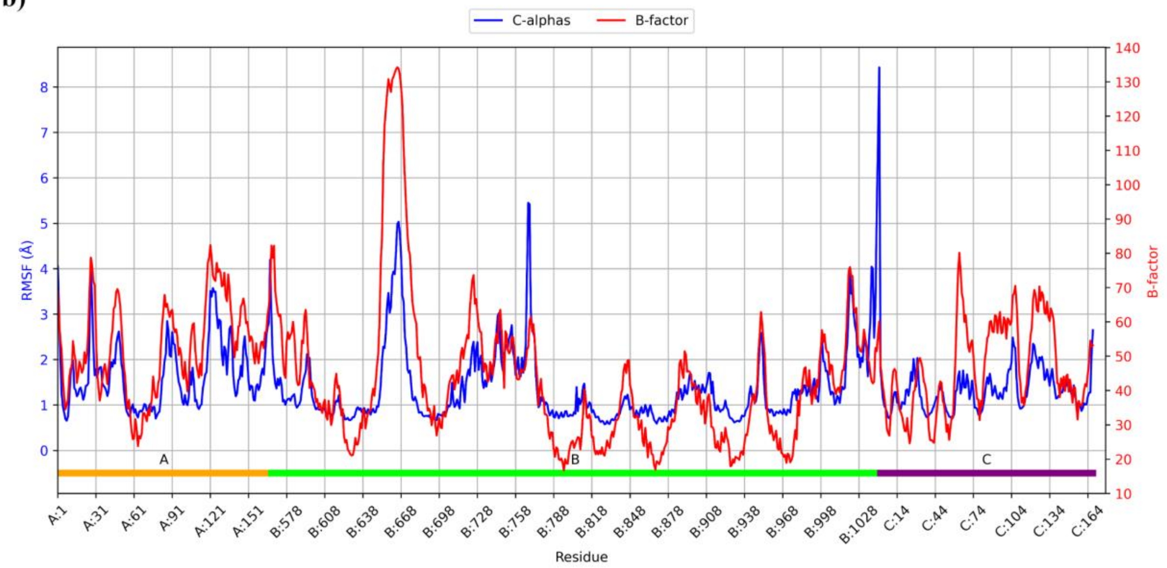

Figure S4. a) Protein RMSD and **b)** Protein RMSF plot of the ternary KRASG13D-SOS1 structure without ligand obtained from 1 μs MD simulation trajectory.

3.2. Compounds Docked at the Ligand Binding Site

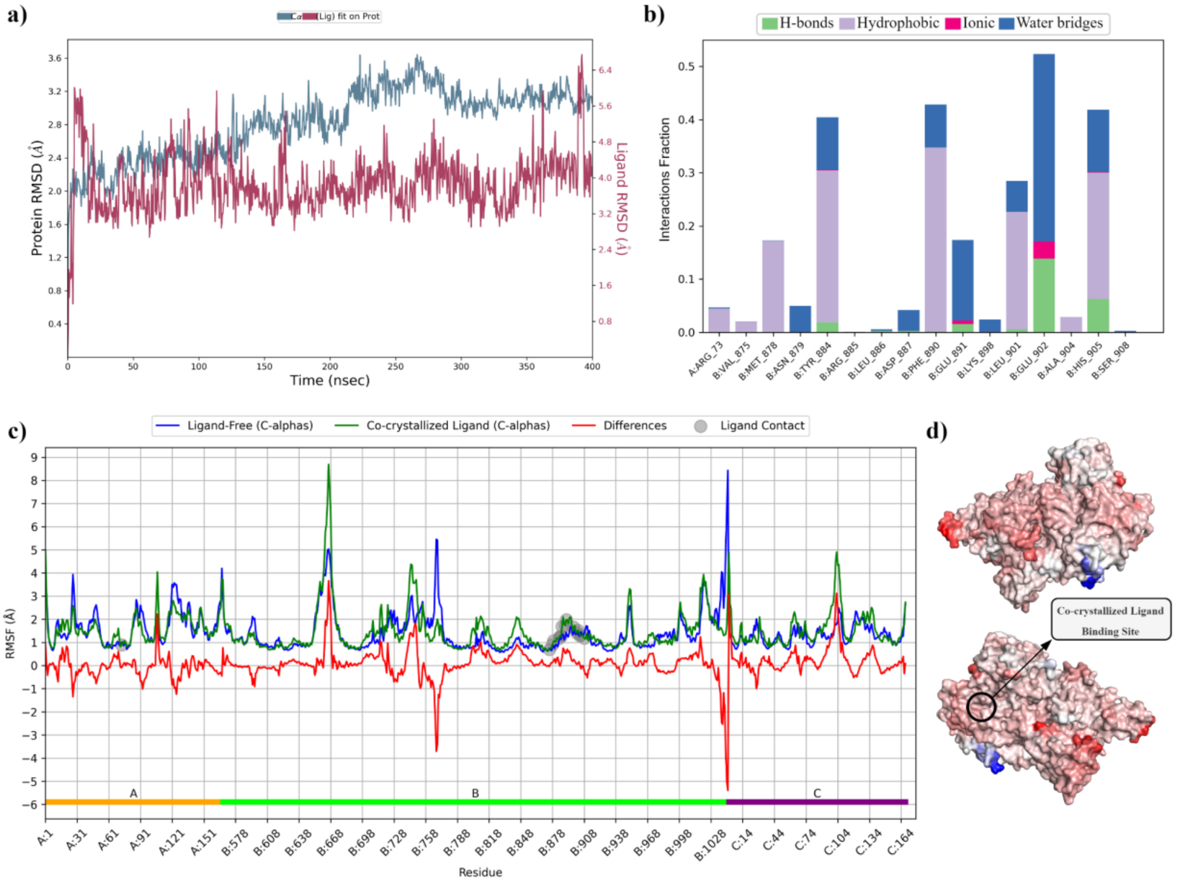

Figure S5. a) Protein and ligand RMSD and **b)** protein-ligand contact histogram of replica-1 of the ternary KRASG13D-SOS1 in complex with co-crystallized ligand BQ5 at co-crystallized ligand binding site obtained from 400 ns MD simulation trajectory **c)** RMSF CA Difference of the ternary complex in complex with co-crystallized ligand BQ5 of replica-1 of MD and the ternary complex without ligand. **d)** The ternary structure viewed from two different perspectives, color-coded by RMSF CA Difference from red (highest) to blue (lowest).

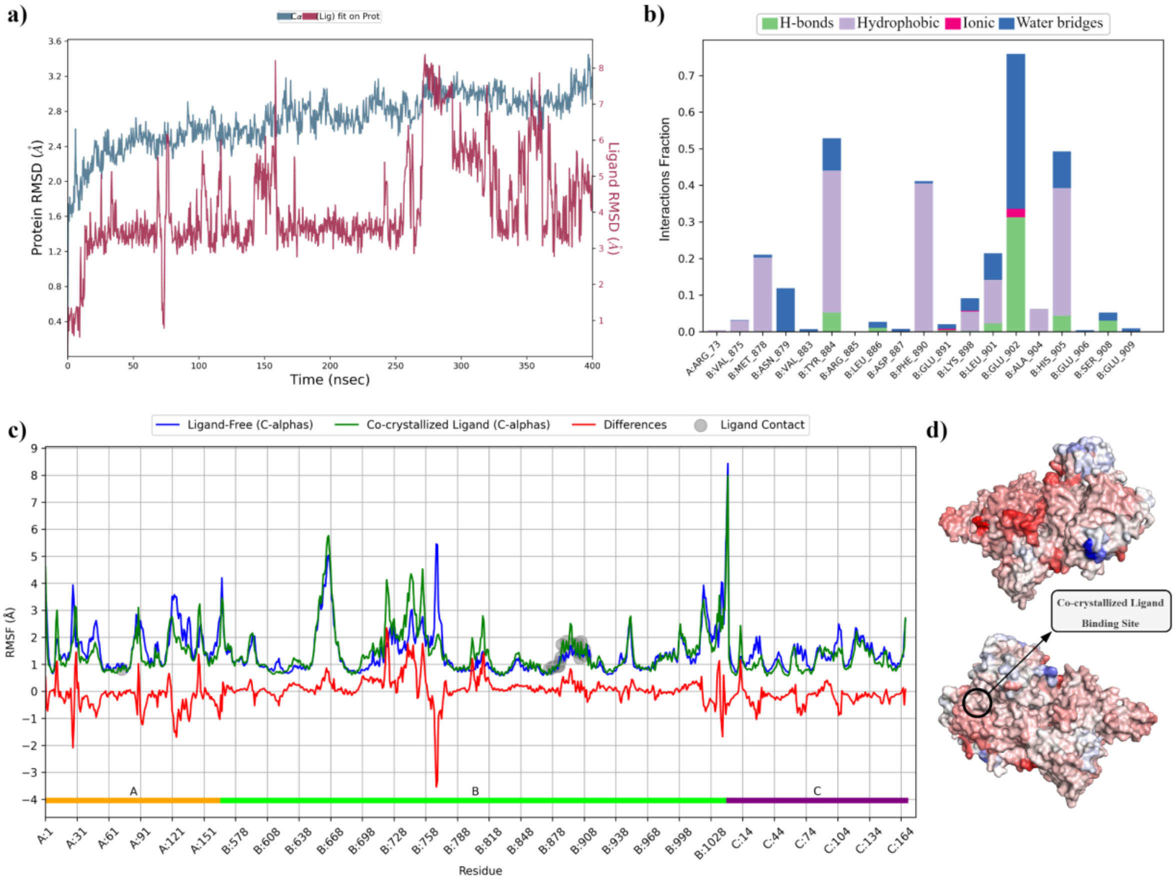

Figure S6. a) Protein and ligand RMSD and **b)** protein-ligand contact histogram of replica-2 of the ternary KRASG13D-SOS1(PDB ID: 7kfz) in complex with co-crystallized ligand BQ5 at co-crystallized ligand binding site obtained from 400 ns MD simulation trajectory **c)** RMSF CA Difference of the ternary complex in complex with co-crystallized ligand BQ5 of replica-2 of MD and the ternary complex without ligand. **d)** The ternary structure viewed from two different perspectives, color-coded by RMSF CA Difference from red (highest) to blue.

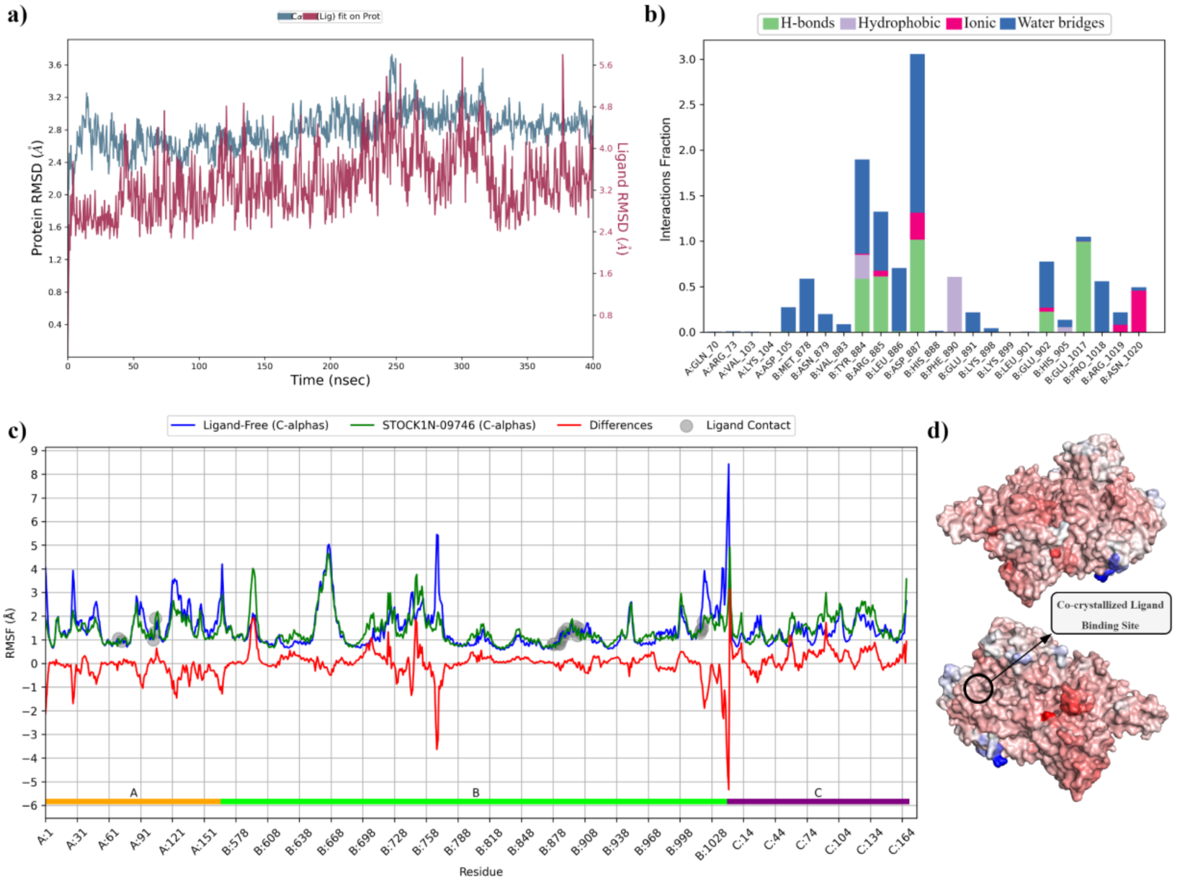

Figure S7. a) Protein and ligand RMSD and **b)** protein-ligand contact histogram of replica-1 of the ternary KRASG13D-SOS1(PDB ID: 7kfz) in complex with STOCK1N-09746 at the co-crystallized ligand binding site obtained from 400 ns MD simulation trajectory **c)** RMSF CA Difference of the ternary complex in complex with STOCK1N-09746 of replica-1 of MD and the ternary complex without ligand. **d)** The ternary structure viewed from two different perspectives, color-coded by RMSF CA Difference from red (highest) to blue (lowest).

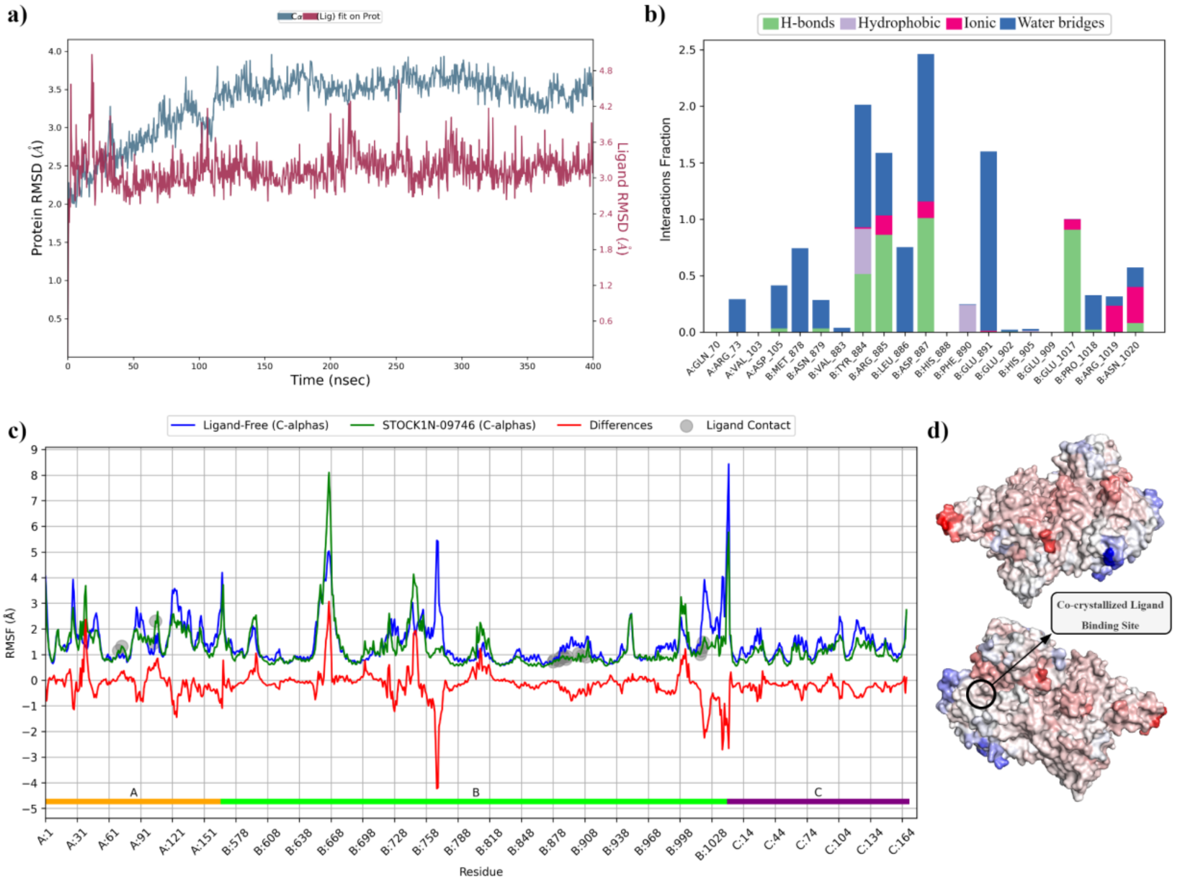

Figure S8. a) Protein and ligand RMSD and **b)** protein-ligand contact histogram of replica-2 of the ternary KRASG13D-SOS1(PDB ID: 7kfz) in complex with STOCK1N-09746 at the co-crystallized ligand binding site obtained from 400 ns MD simulation trajectory **c)** RMSF CA Difference of the ternary complex in complex with STOCK1N-09746 of replica-2 of MD and the ternary complex without ligand. **d)** The ternary structure viewed from two different perspectives, color-coded by RMSF CA Difference from red (highest) to blue (lowest).

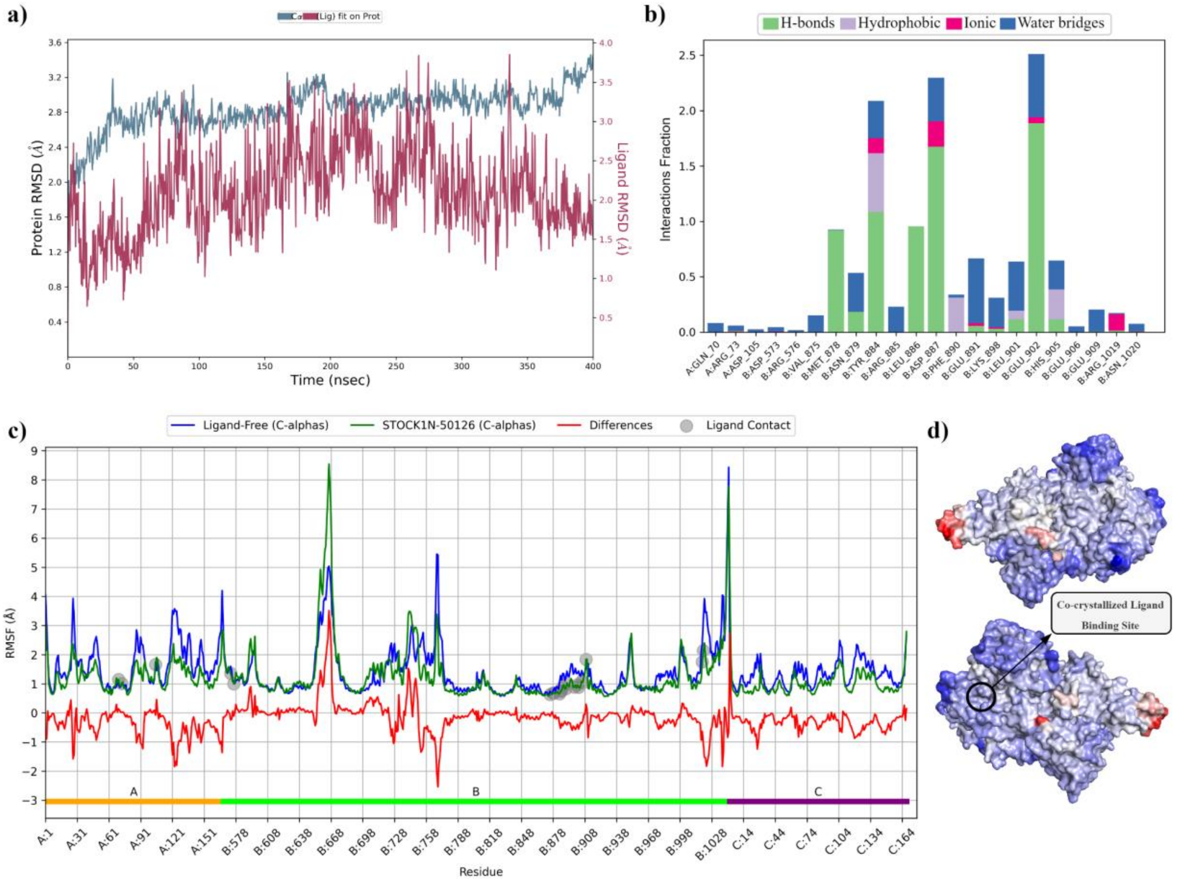

Figure S9. a) Protein and ligand RMSD and **b)** protein-ligand contact histogram of replica-1 of the ternary KRASG13D-SOS1(PDB ID: 7kfz) in complex with STOCK1N-50126 at the co-crystallized ligand binding site obtained from 400 ns MD simulation trajectory **c)** RMSF CA Difference of the ternary complex in complex with STOCK1N-50126 of replica-1 of MD and the ternary complex without ligand. **d)** The ternary structure viewed from two different perspectives, color-coded by RMSF CA Difference from red (highest) to blue (lowest).

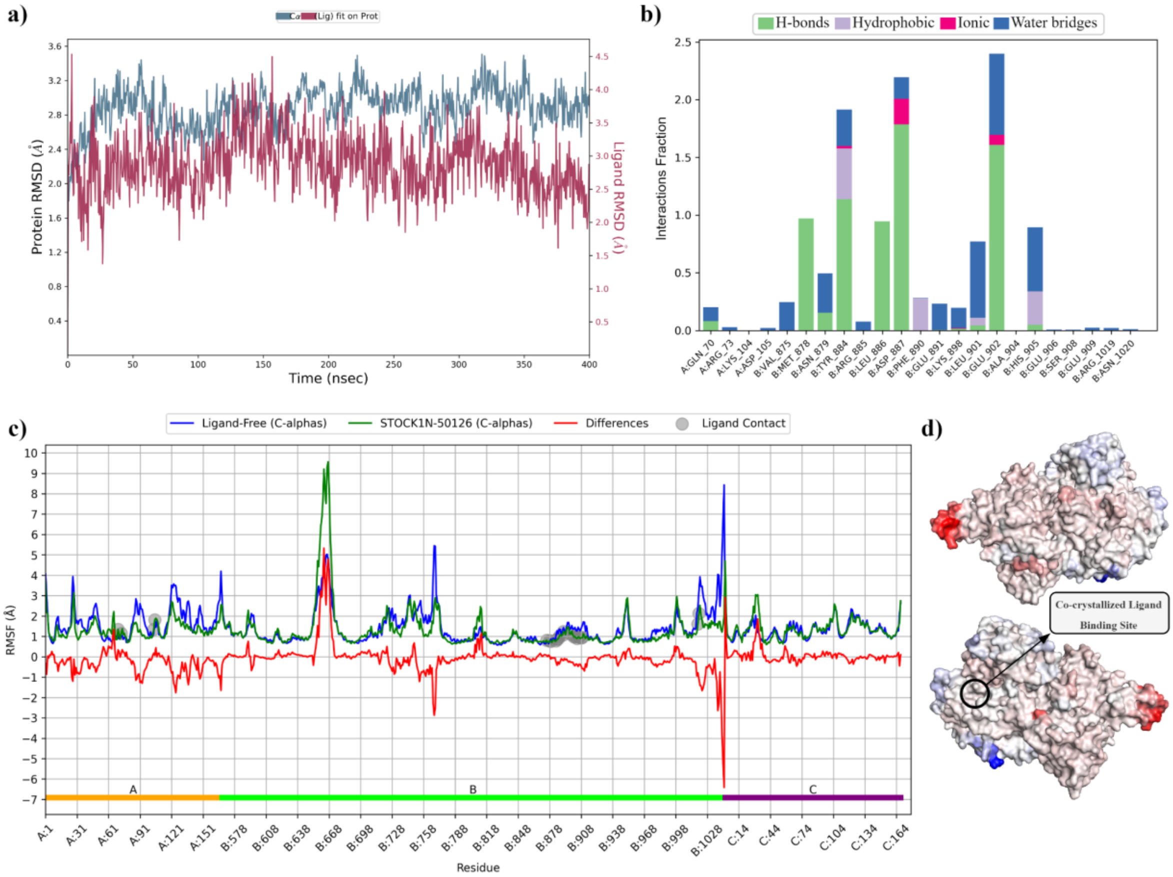

Figure S10. a) Protein and ligand RMSD and **b)** protein-ligand contact histogram of replica-2 of the ternary KRASG13D-SOS1(PDB ID: 7kfz) in complex with STOCK1N-50126 at the co-crystallized ligand binding site obtained from 400 ns MD simulation trajectory **c)** RMSF CA Difference of the ternary complex in complex with STOCK1N-50126 of replica-2 of MD and the ternary complex without ligand. **d)** The ternary structure viewed from two different perspectives, color-coded by RMSF CA Difference from red (highest) to blue (lowest).

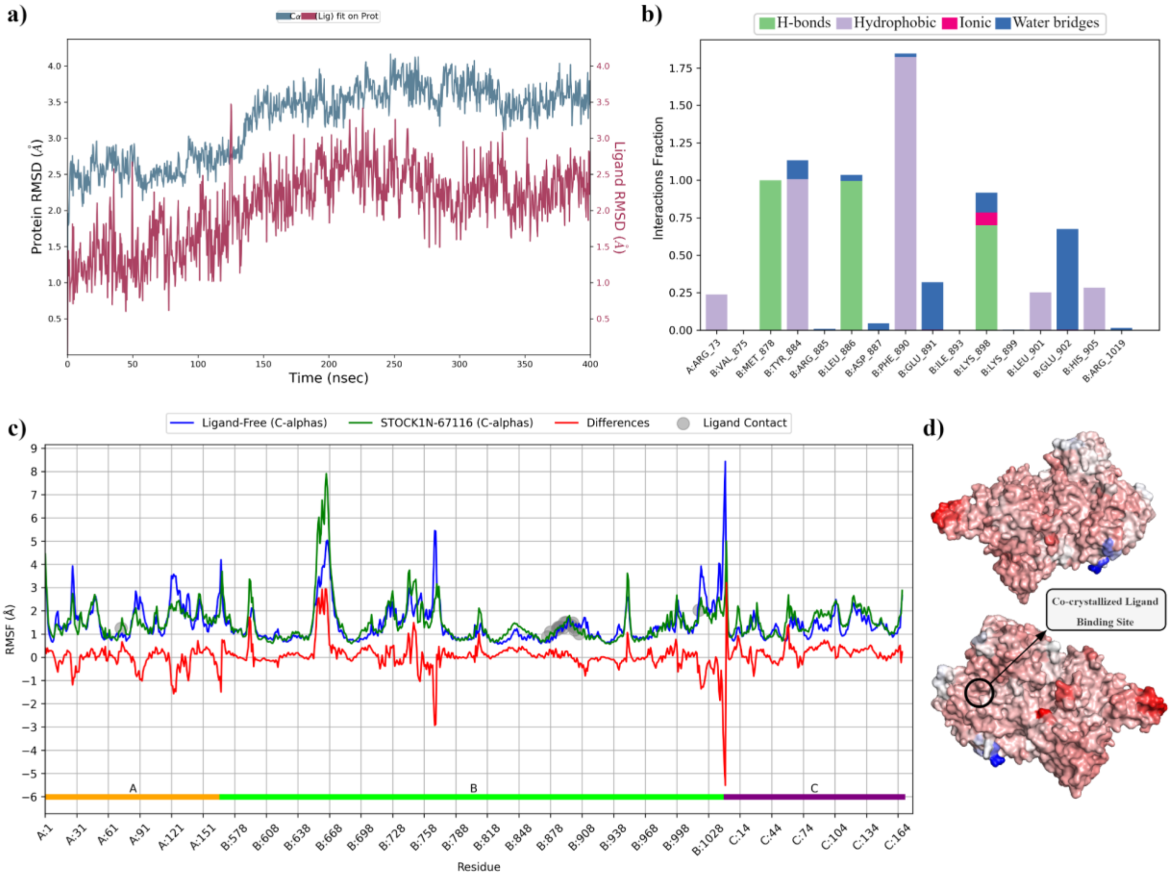

Figure S11.a) Protein and ligand RMSD and **b)** protein-ligand contact histogram of replica-1 of the ternary KRASG13D-SOS1(PDB ID: 7kfz) in complex with STOCK1N-67116 at the co-crystallized ligand binding site obtained from 400 ns MD simulation trajectory **c)** RMSF CA Difference of the ternary complex in complex with STOCK1N-67116 of replica-1 of MD and the ternary complex without ligand. **d)** The ternary structure viewed from two different perspectives, color-coded by RMSF CA Difference from red (highest) to blue (lowest).

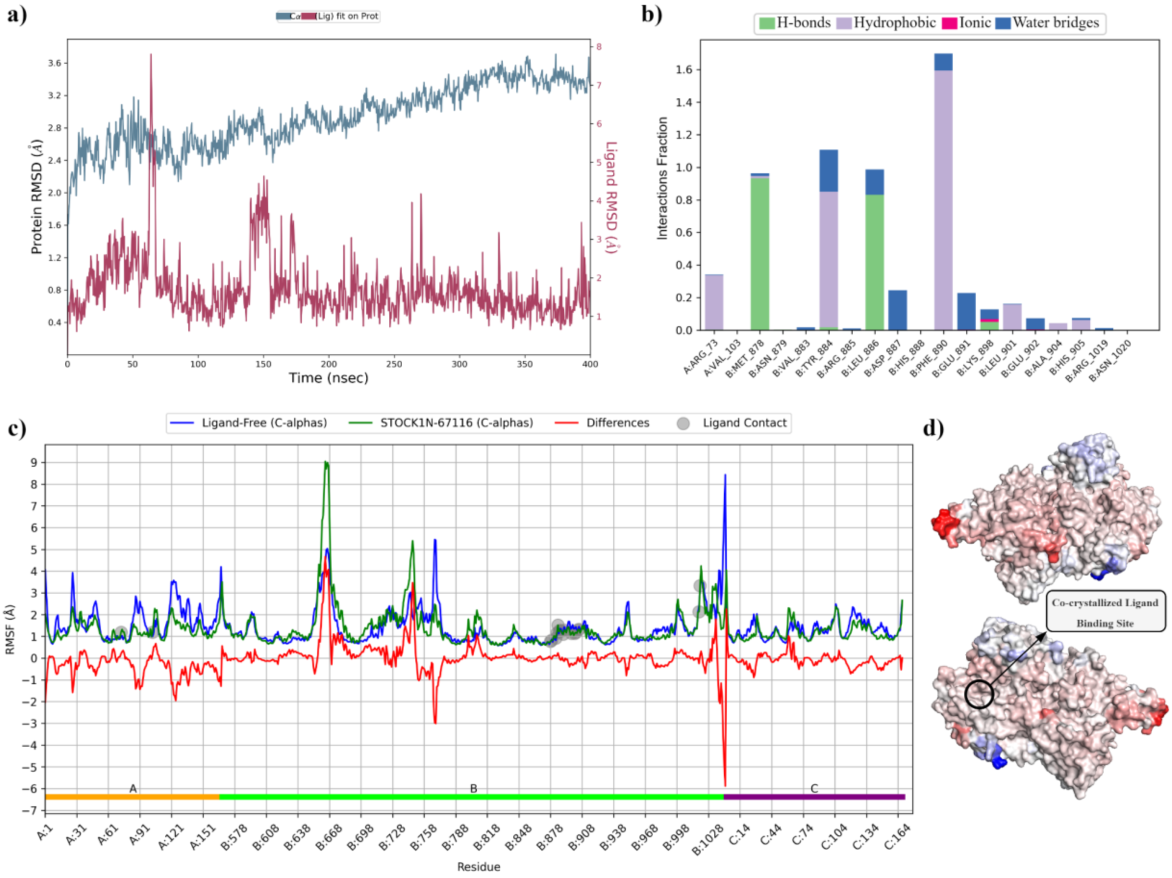

Figure S12. a) Protein and ligand RMSD and **b)** protein-ligand contact histogram of replica-2 of the ternary KRASG13D-SOS1(PDB ID: 7kfz) in complex with STOCK1N-67116 at the co-crystallized ligand binding site obtained from 400 ns MD simulation trajectory **c)** RMSF CA Difference of the ternary complex in complex with STOCK1N-67116 of replica-2 of MD and the ternary complex without ligand. **d)** The ternary structure viewed from two different perspectives, color-coded by RMSF CA Difference from red (highest) to blue (lowest).

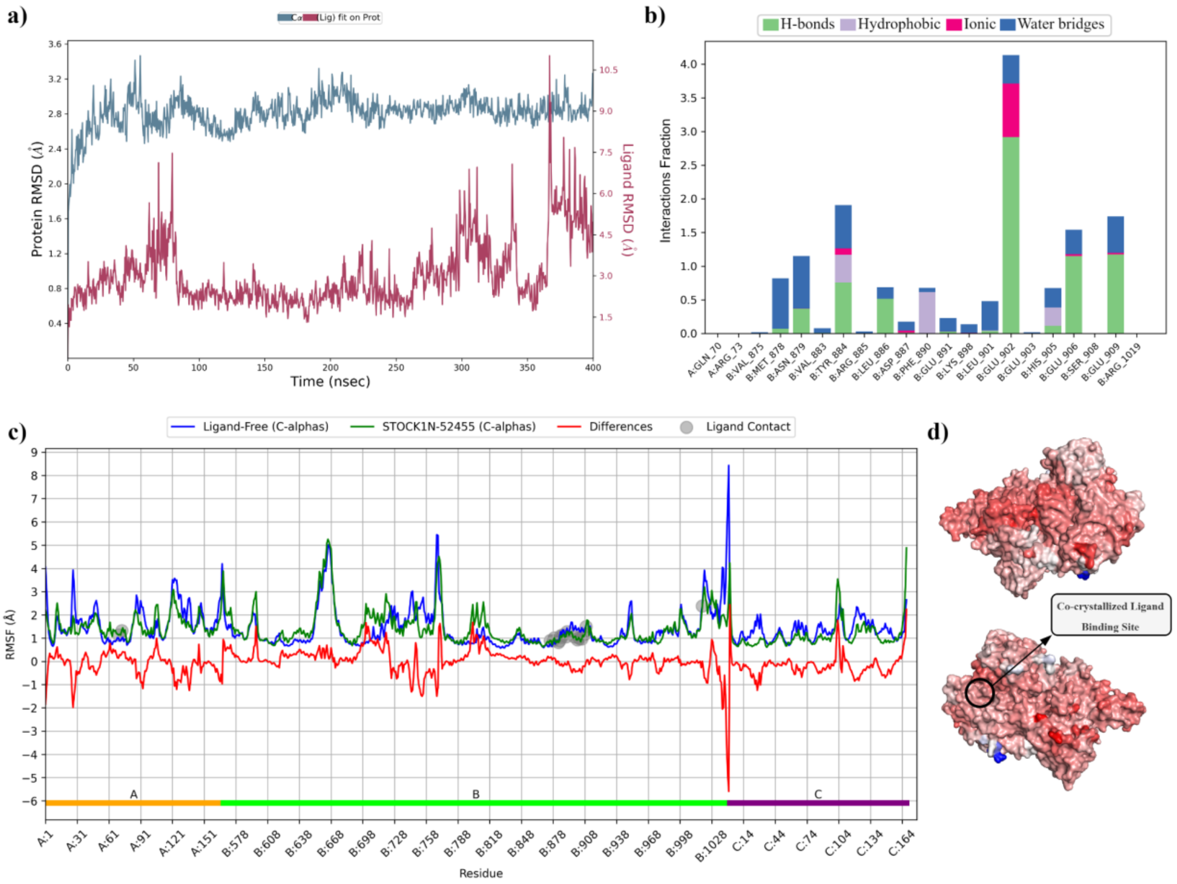

Figure S13. a) Protein and ligand RMSD and **b)** protein-ligand contact histogram of replica-1 of the ternary KRASG13D-SOS1(PDB ID: 7kfz) in complex with STOCK1N-52455 at the co-crystallized ligand binding site obtained from 400 ns MD simulation trajectory **c)** RMSF CA Difference of the ternary complex in complex with STOCK1N-52455 of replica-1 of MD and the ternary complex without ligand. **d)** The ternary structure viewed from two different perspectives, color-coded by RMSF CA Difference from red (highest) to blue (lowest).

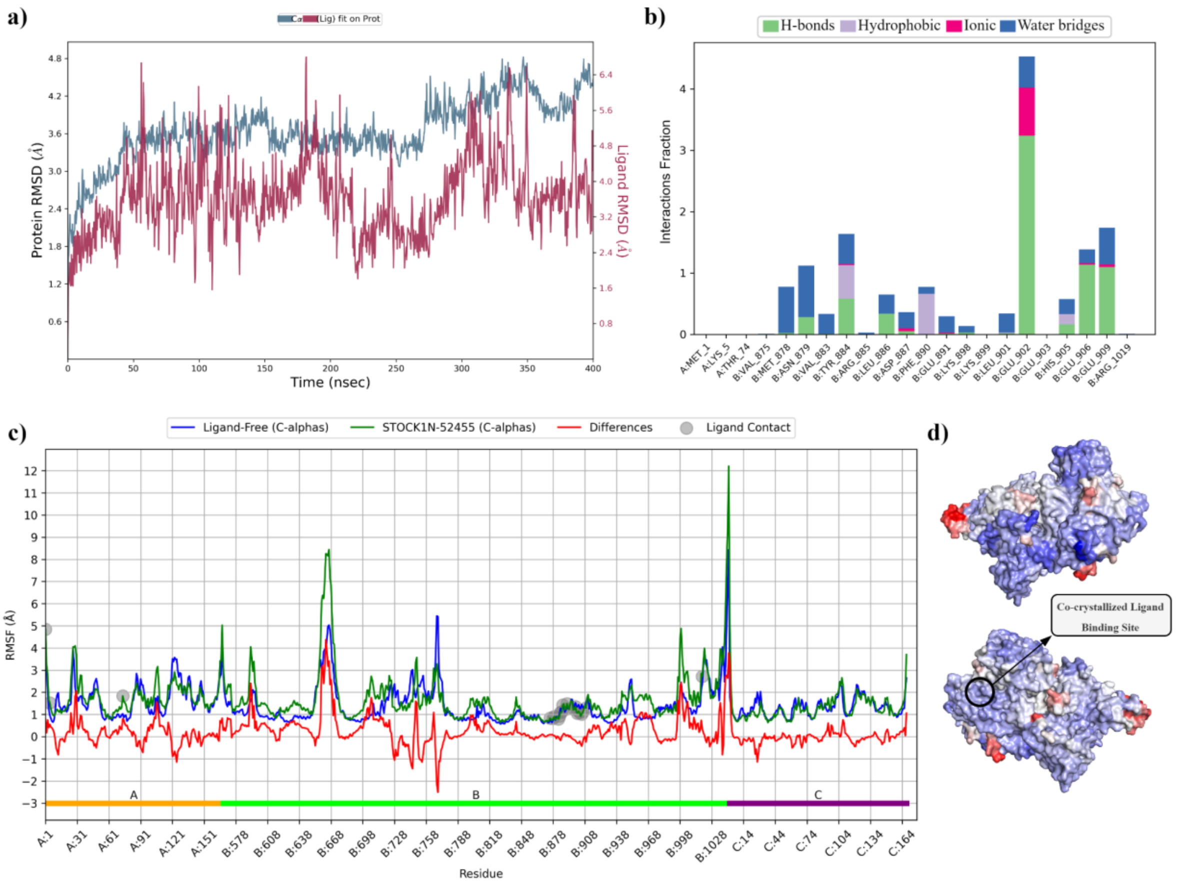

Figure S14. a) Protein and ligand RMSD and **b)** protein-ligand contact histogram of replica-2 of the ternary KRASG13D-SOS1(PDB ID: 7kfz) in complex with STOCK1N-52455 at the co-crystallized ligand binding site obtained from 400 ns MD simulation trajectory **c)** RMSF CA Difference of the ternary complex in complex with STOCK1N-52455 of replica-2 of MD and the ternary complex without ligand. **d)** The ternary structure viewed from two different perspectives, color-coded by RMSF CA Difference from red (highest) to blue (lowest).

3.3. Compounds Docked at the Allosteric Pocket P1

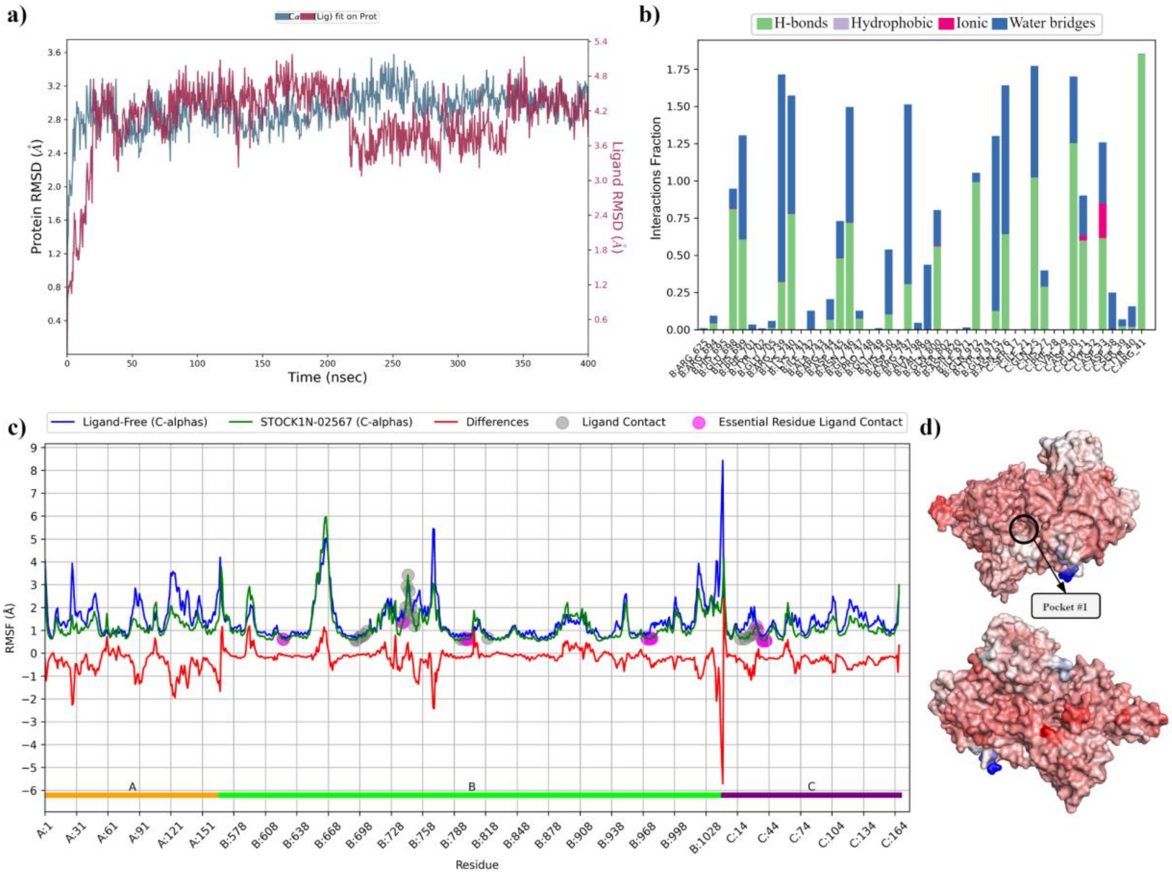

Figure S15. a) Protein and ligand RMSD and **b)** protein-ligand contact histogram of replica-1 of the ternary KRASG13D-SOS1(PDB ID: 7kfz) in complex with STOCK1N-02567 at potential allosteric pocket #1 site obtained from 400 ns MD simulation trajectory **c)** RMSF CA Difference of the ternary complex in complex with STOCK1N-02657 of replica-1 of MD and the ternary complex without ligand. **d)** The ternary structure viewed from two different perspectives, color-coded by RMSF CA Difference from red (highest) to blue (lowest).

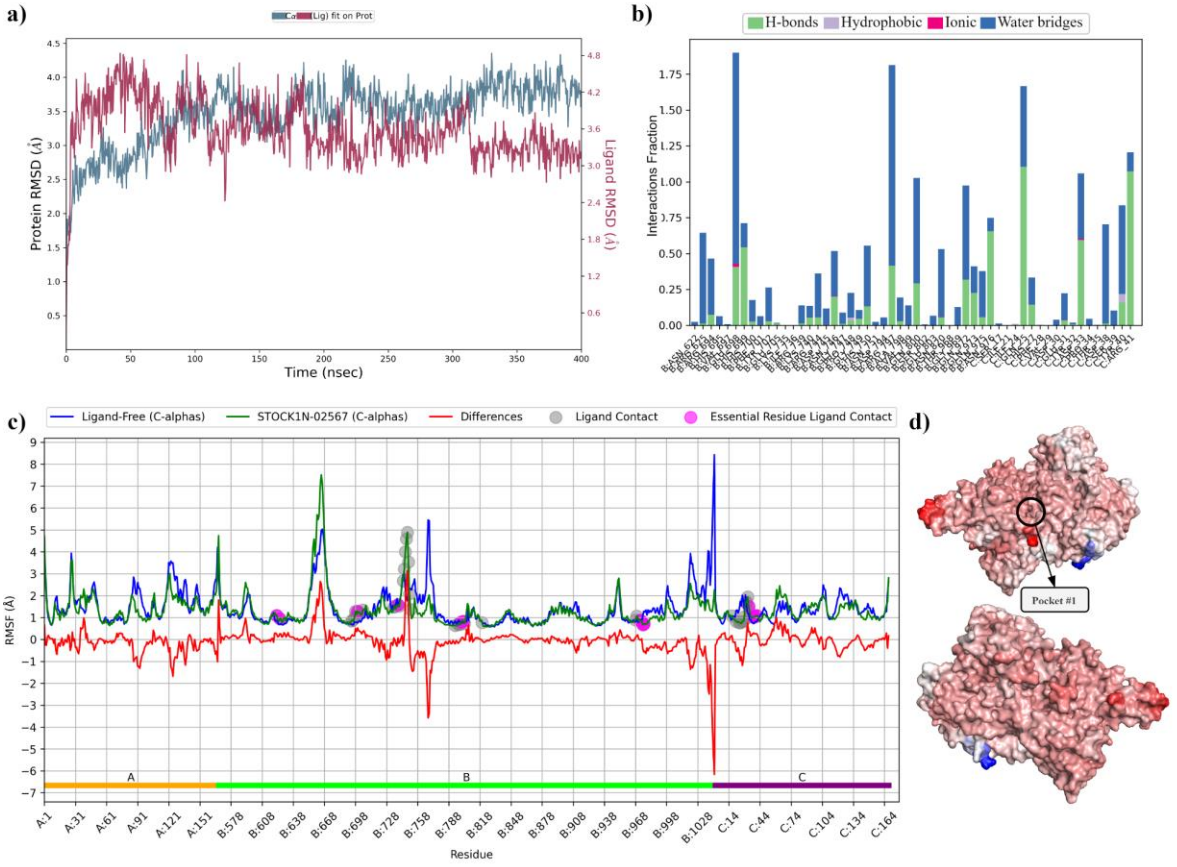

Figure S16. a) Protein and ligand RMSD and **b)** protein-ligand contact histogram of replica-2 of the ternary KRASG13D-SOS1(PDB ID: 7kfz) in complex with STOCK1N-02567 at potential allosteric pocket #1 site obtained from 400 ns MD simulation trajectory **c)** RMSF CA Difference of the ternary complex in complex with STOCK1N-02657 of replica-2 of MD and the ternary complex without ligand. **d)** The ternary structure viewed from two different perspectives, color-coded by RMSF CA Difference from red (highest) to blue (lowest).

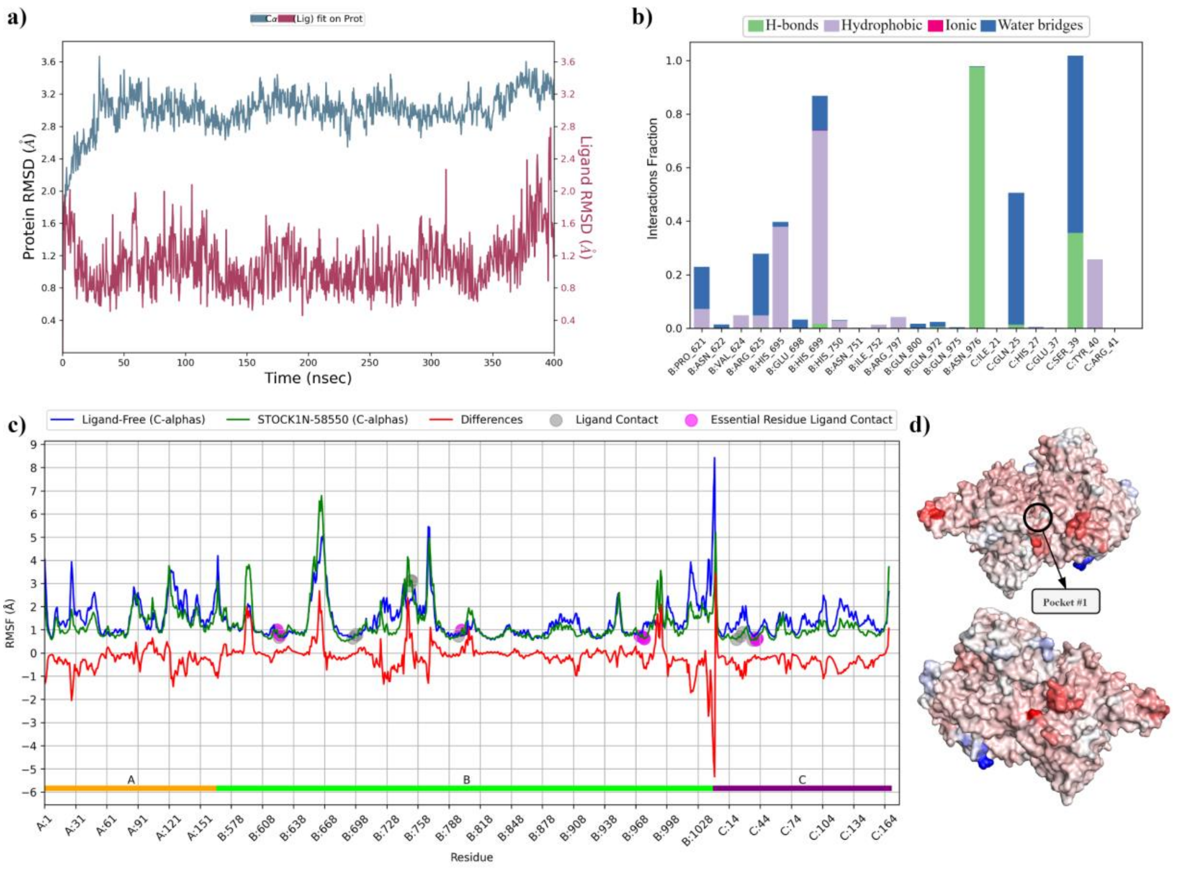

Figure S17. a) Protein and ligand RMSD and **b)** protein-ligand contact histogram of replica-1 of the ternary KRASG13D-SOS1(PDB ID: 7kfz) in complex with STOCK1N-58550 at potential allosteric pocket #1 site obtained from 400 ns MD simulation trajectory **c)** RMSF CA Difference of the ternary complex in complex with STOCK1N-58550 of replica-1 of MD and the ternary complex without ligand. **d)** The ternary structure viewed from two different perspectives, color-coded by RMSF CA Difference from red (highest) to blue (lowest).

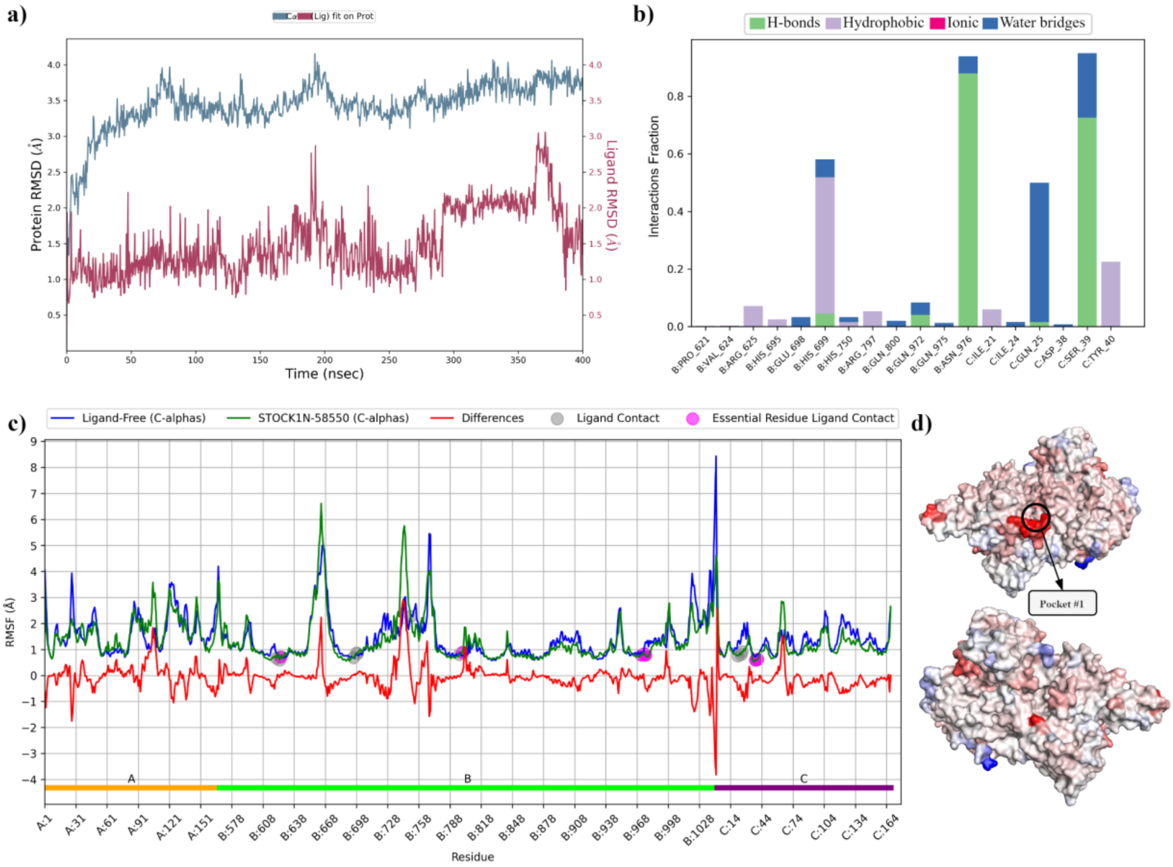

Figure S18. a) Protein and ligand RMSD and **b)** protein-ligand contact histogram of replica-2 of the ternary KRASG13D-SOS1(PDB ID: 7kfz) in complex with STOCK1N-58550 at potential allosteric pocket #1 site obtained from 400 ns MD simulation trajectory **c)** RMSF CA Difference of the ternary complex in complex with STOCK1N-58550 of replica-2 of MD and the ternary complex without ligand. **d)** The ternary structure viewed from two different perspectives, color-coded by RMSF CA Difference from red (highest) to blue (lowest).

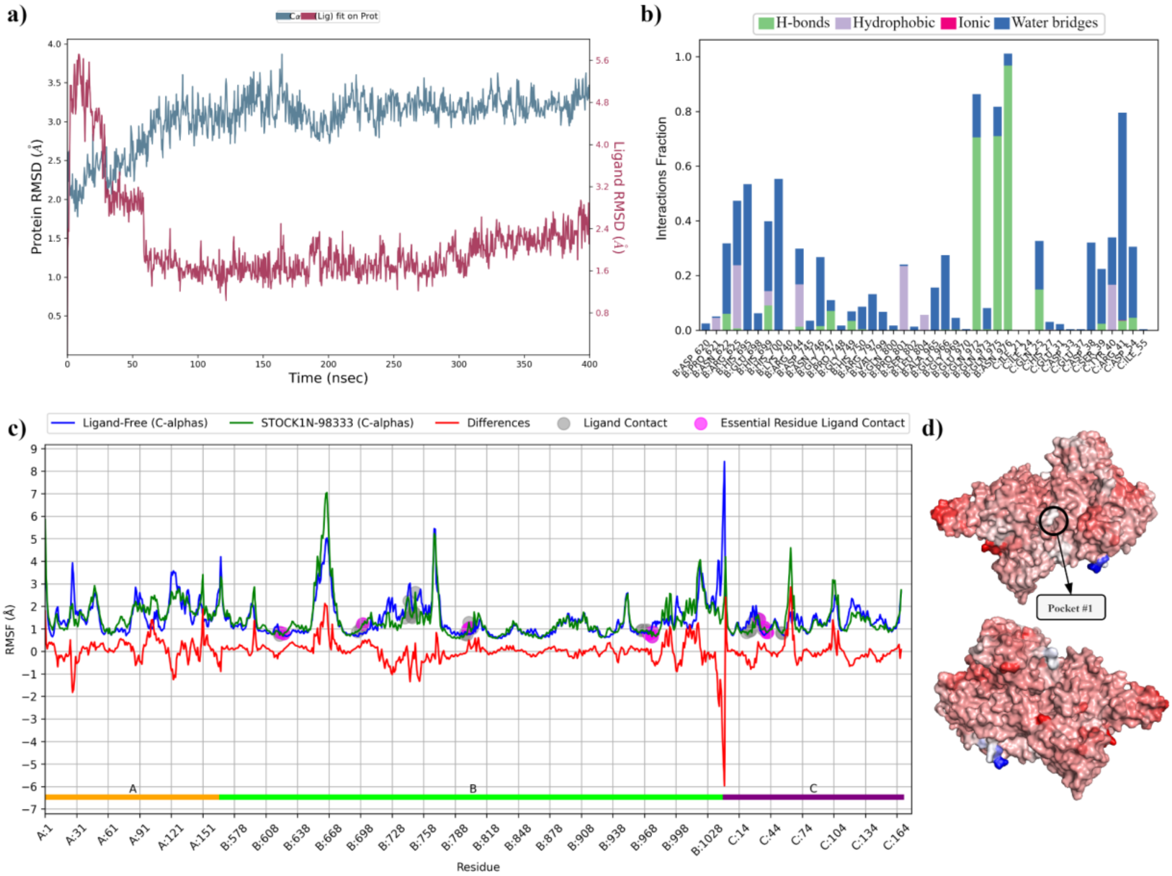

Figure S19. a) Protein and ligand RMSD and **b)** protein-ligand contact histogram of replica-1 of the ternary KRASG13D-SOS1(PDB ID: 7kfz) in complex with STOCK1N-98333 at potential allosteric pocket #1 site obtained from 400 ns MD simulation trajectory **c)** RMSF CA Difference of the ternary complex in complex with STOCK1N-98333 of replica-1 of MD and the ternary complex without ligand. **d)** The ternary structure viewed from two different perspectives, color-coded by RMSF CA Difference from red (highest) to blue (lowest).

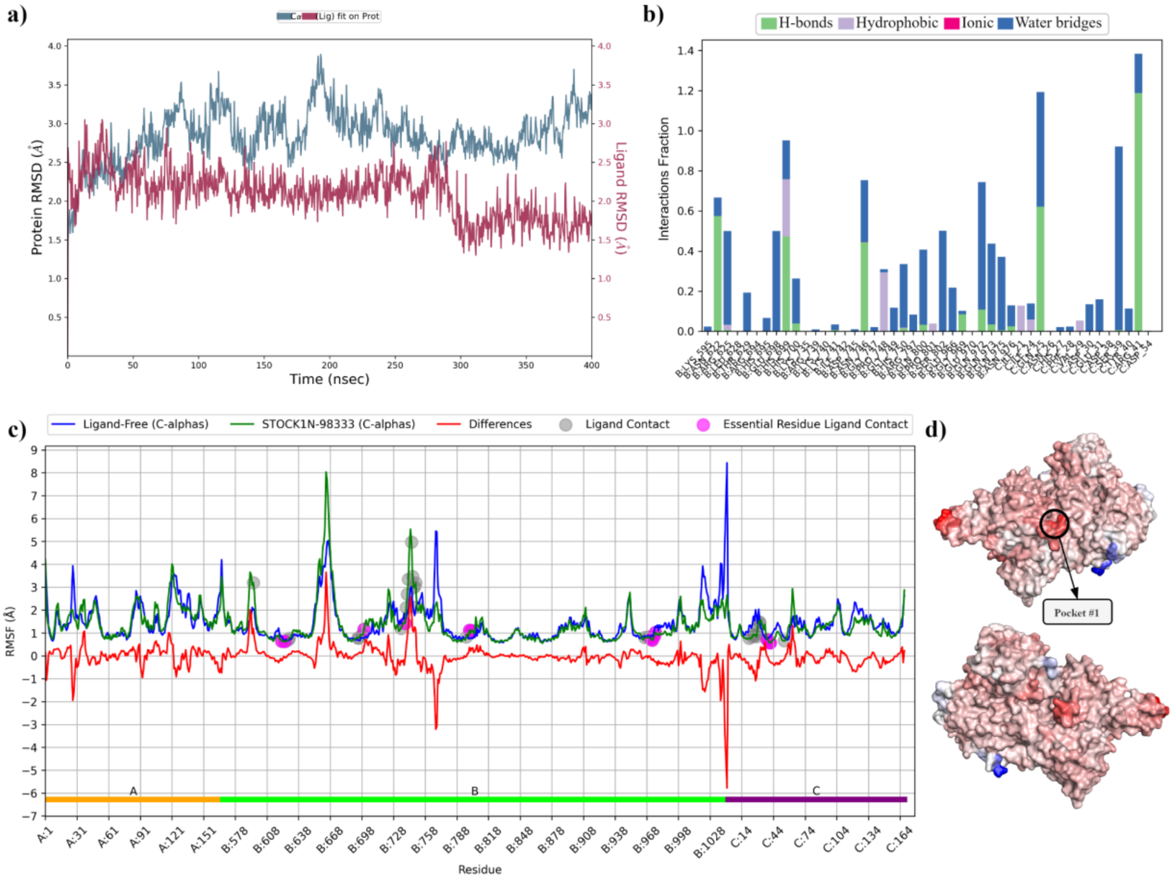

Figure S20. a) Protein and ligand RMSD and **b)** protein-ligand contact histogram of replica-2 of the ternary KRASG13D-SOS1(PDB ID: 7kfz) in complex with STOCK1N-98333 at potential allosteric pocket #1 site obtained from 400 ns MD simulation trajectory **c)** RMSF CA Difference of the ternary complex in complex with STOCK1N-98333 of replica-2 of MD and the ternary complex without ligand. **d)** The ternary structure viewed from two different perspectives, color-coded by RMSF CA Difference from red (highest) to blue (lowest).

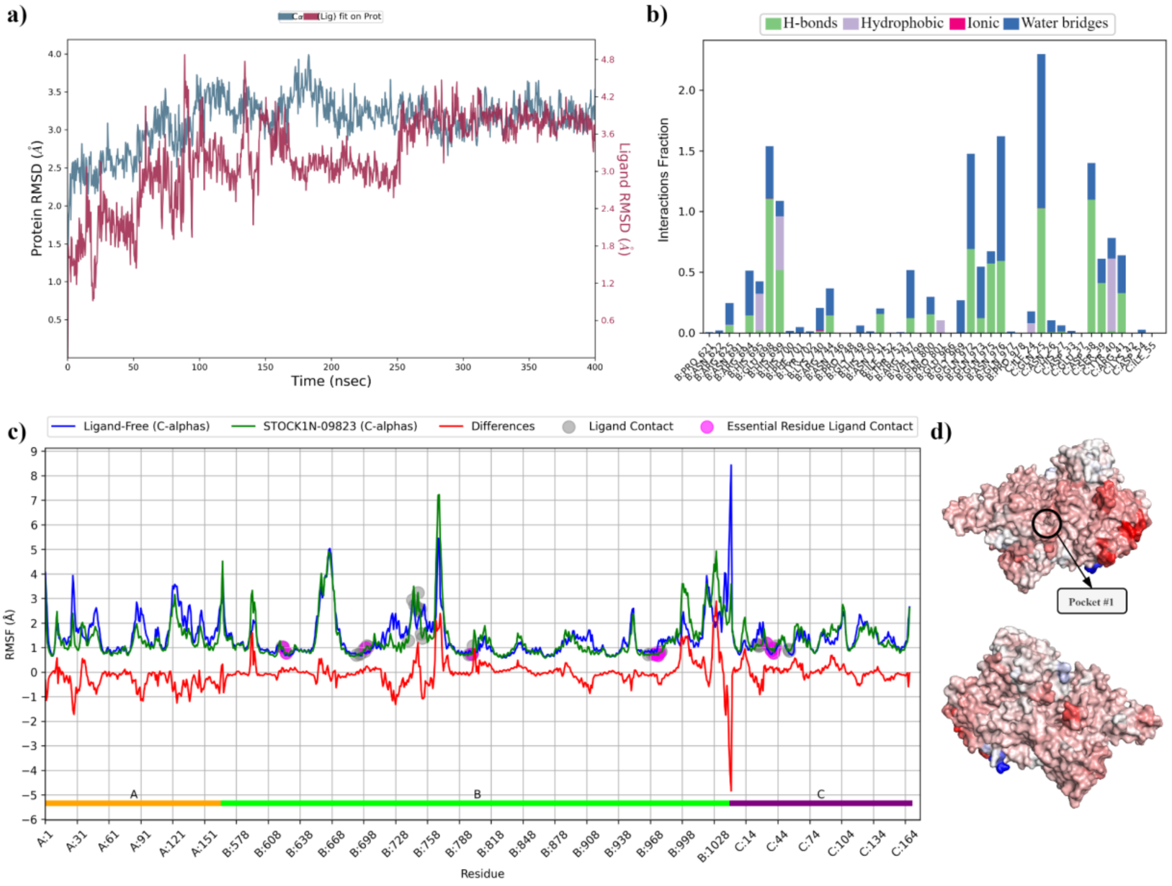

Figure S21. **a)** Protein and ligand RMSD and **b)** protein-ligand contact histogram of replica-1 of the ternary KRASG13D-SOS1(PDB ID: 7kfz) in complex with STOCK1N-09823 at potential allosteric pocket #1 site obtained from 400 ns MD simulation trajectory **c)** RMSF CA Difference of the ternary complex in complex with STOCK1N-09823 of replica-1 of MD and the ternary complex without ligand. **d)** The ternary structure viewed from two different perspectives, color-coded by RMSF CA Difference from red (highest) to blue (lowest).

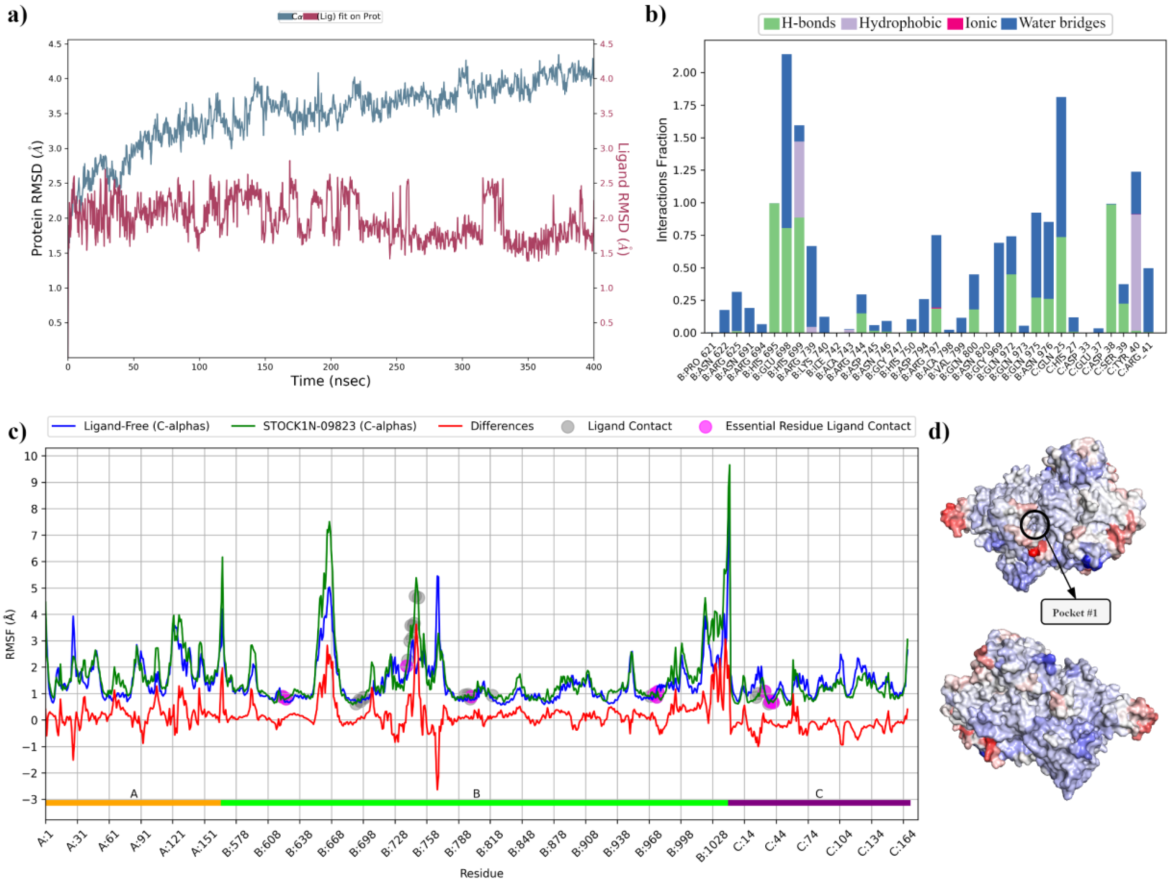

Figure S22. **a)** Protein and ligand RMSD and **b)** protein-ligand contact histogram of replica-2 of the ternary KRASG13D-SOS1(PDB ID: 7kfz) in complex with STOCK1N-09823 at potential allosteric pocket #1 site obtained from 400 ns MD simulation trajectory **c)** RMSF CA Difference of the ternary complex in complex with STOCK1N-09823 of replica-2 of MD and the ternary complex without ligand. **d)** The ternary structure viewed from two different perspectives, color-coded by RMSF CA Difference from red (highest) to blue (lowest).

3.4. Compounds Docked at the Allosteric Pocket P2

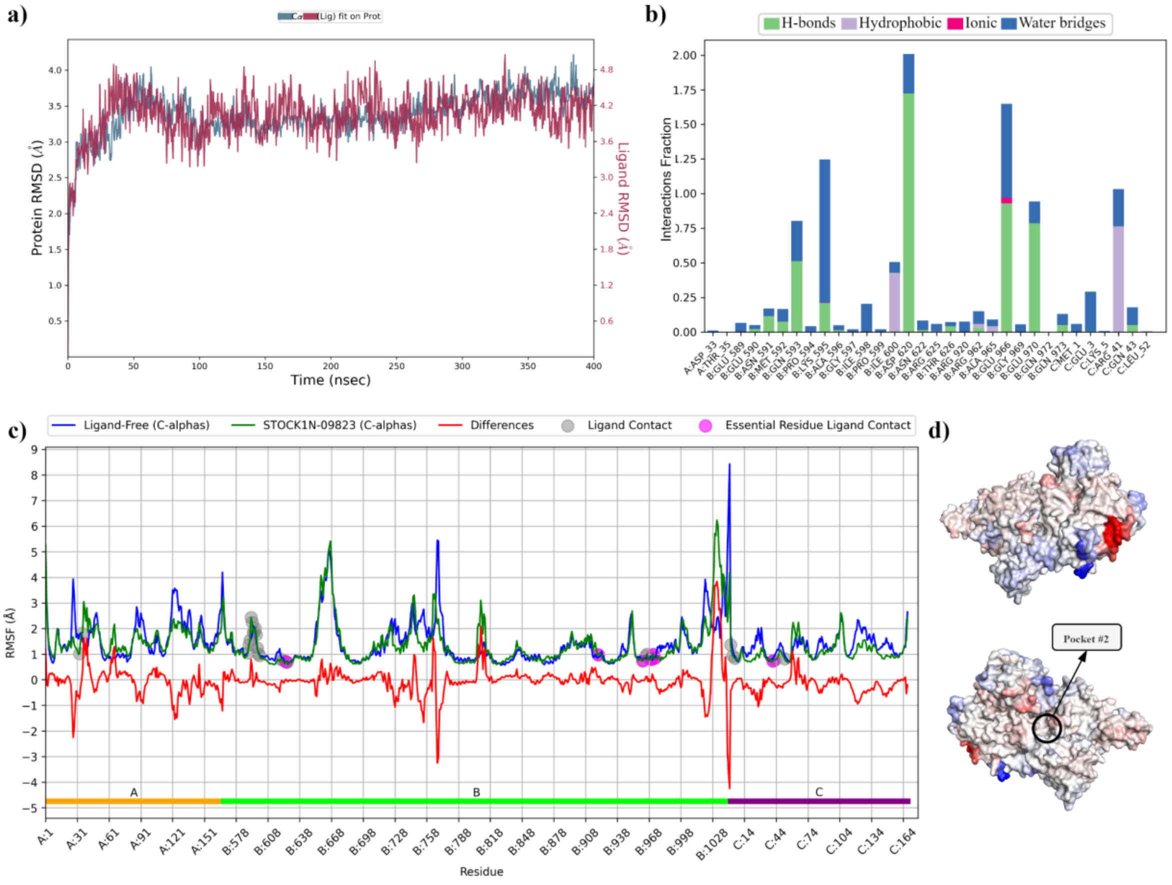

Figure S23. a) Protein and ligand RMSD and **b)** protein-ligand contact histogram of replica-1 of the ternary KRASG13D-SOS1(PDB ID: 7kfz) in complex with STOCK1N-09823 at potential allosteric pocket #2 site obtained from 400 ns MD simulation trajectory **c)** RMSF CA Difference of the ternary complex in complex with STOCK1N-09823 of replica-1 of MD and the ternary complex without ligand. **d)** The ternary structure viewed from two different perspectives, color-coded by RMSF CA Difference from red (highest) to blue (lowest).

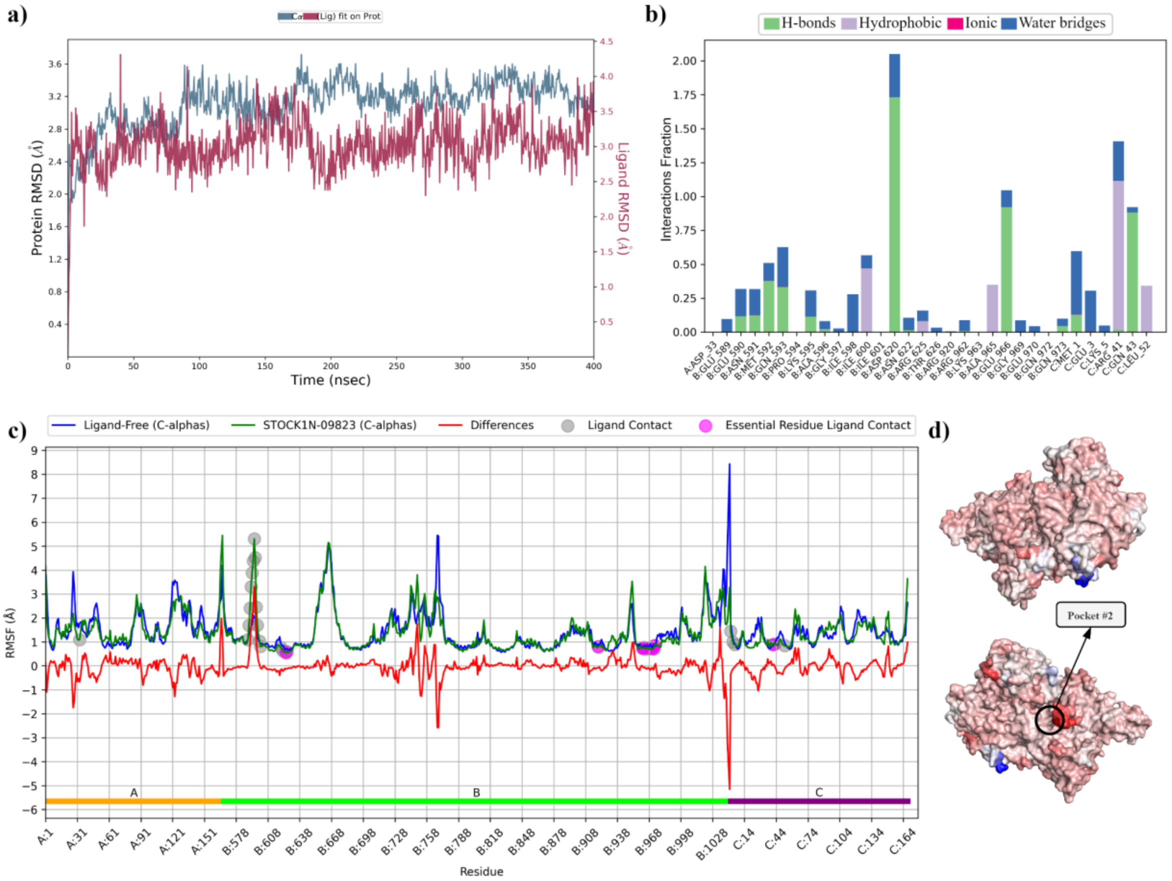

Figure S24. a) Protein and ligand RMSD and **b)** protein-ligand contact histogram of replica-2 of the ternary KRASG13D-SOS1(PDB ID: 7kfz) in complex with STOCK1N-09823 at potential allosteric pocket #2 site obtained from 400 ns MD simulation trajectory **c)** RMSF CA Difference of the ternary complex in complex with STOCK1N-09823 of replica-2 of MD and the ternary complex without ligand. **d)** The ternary structure viewed from two different perspectives, color-coded by RMSF CA Difference from red (highest) to blue (lowest).

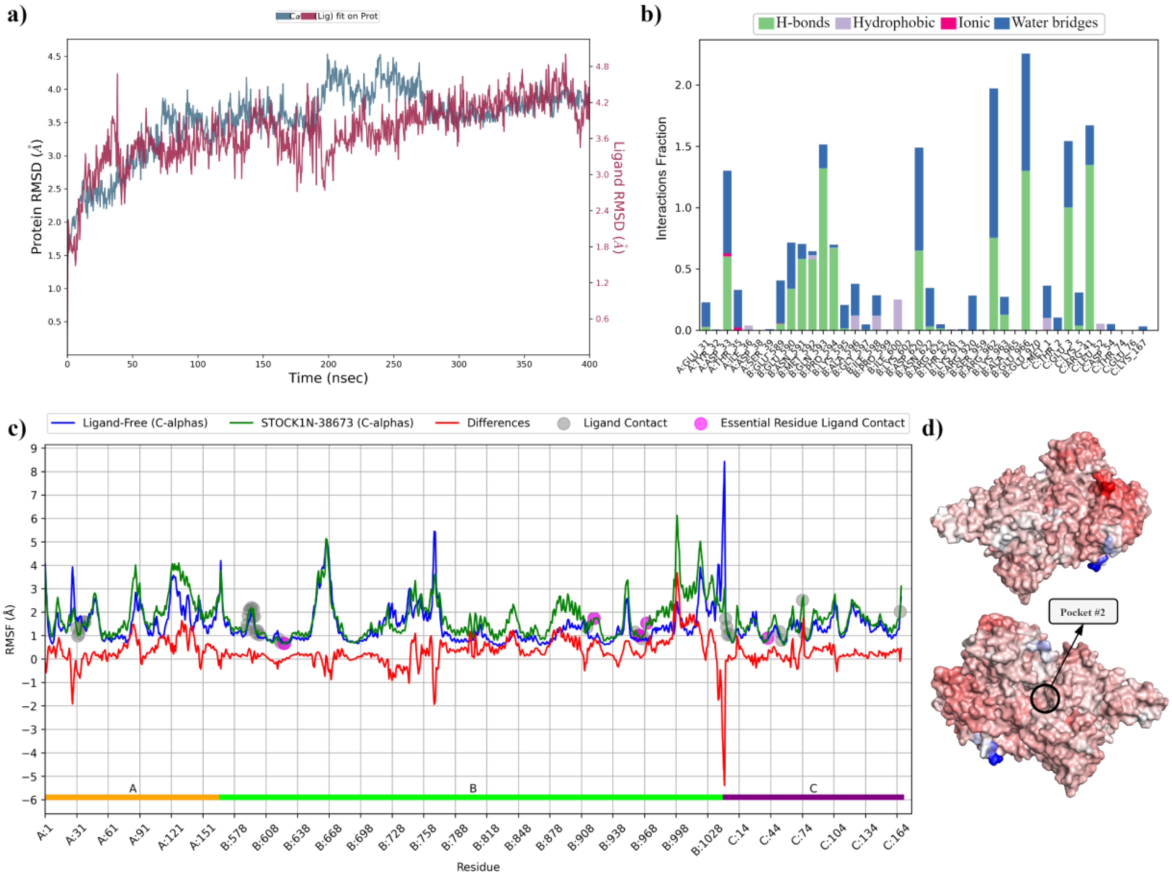

Figure S25. a) Protein and ligand RMSD and **b)** protein-ligand contact histogram of replica-1 of the ternary KRASG13D-SOS1(PDB ID: 7kfz) in complex with STOCK1N-38673 at potential allosteric pocket #2 site obtained from 400 ns MD simulation trajectory **c)** RMSF CA Difference of the ternary complex in complex with STOCK1N-38673 of replica-1 of MD and the ternary complex without ligand. **d)** The ternary structure viewed from two different perspectives, color-coded by RMSF CA Difference from red (highest) to blue (lowest).

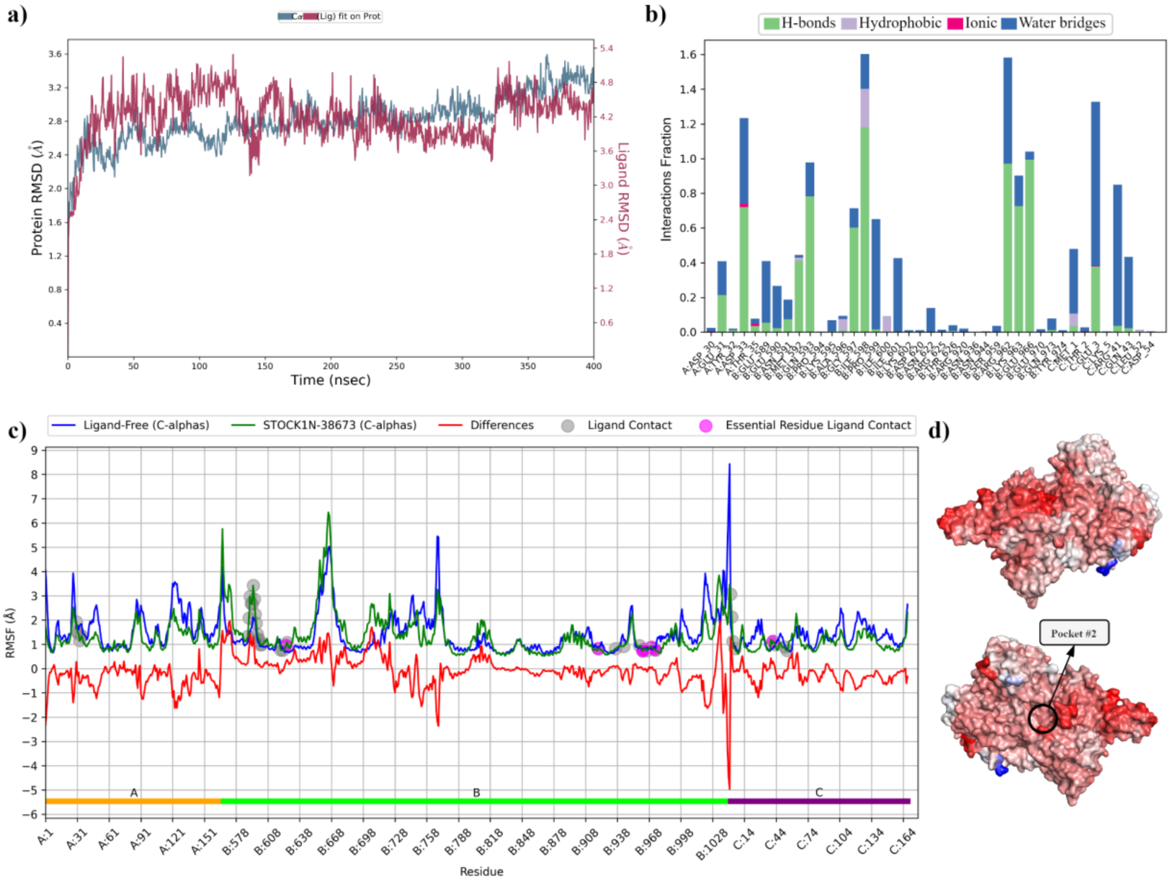

Figure S26. a) Protein and ligand RMSD and **b)** protein-ligand contact histogram of replica-2 of the ternary KRASG13D-SOS1(PDB ID: 7kfz) in complex with STOCK1N-38673 at potential allosteric pocket #2 site obtained from 400 ns MD simulation trajectory **c)** RMSF CA Difference of the ternary complex in complex with STOCK1N-38673 of replica-2 of MD and the ternary complex without ligand. **d)** The ternary structure viewed from two different perspectives, color-coded by RMSF CA Difference from red (highest) to blue (lowest).

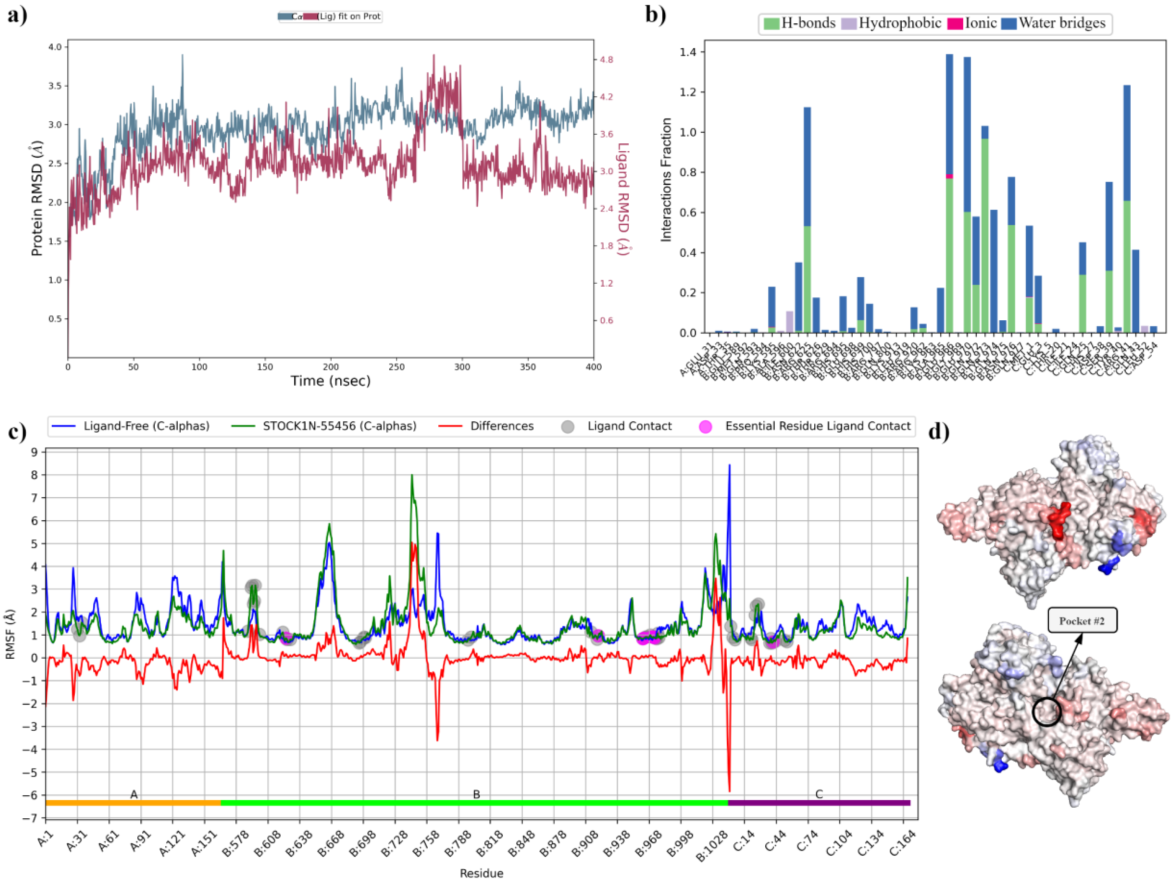

Figure S27. a) Protein and ligand RMSD and **b)** protein-ligand contact histogram of replica-1 of the ternary KRASG13D-SOS1(PDB ID: 7kfz) in complex with STOCK1N-55456 at potential allosteric pocket #2 site obtained from 400 ns MD simulation trajectory **c)** RMSF CA Difference of the ternary complex in complex with STOCK1N-55456 of replica-1 of MD and the ternary complex without ligand. **d)** The ternary structure viewed from two different perspectives, color-coded by RMSF CA Difference from red (highest) to blue (lowest).

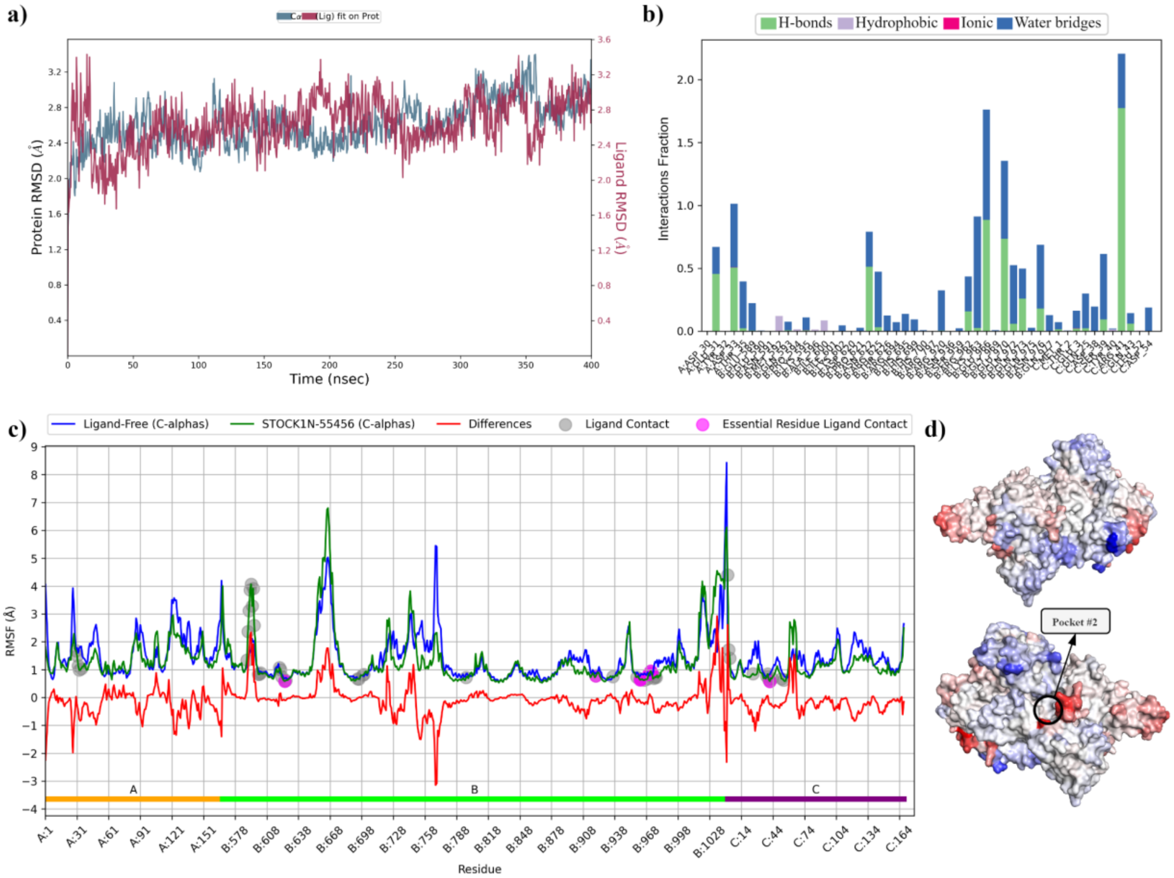

Figure S28. a) Protein and ligand RMSD and **b)** protein-ligand contact histogram of replica-2 of the ternary KRASG13D-SOS1(PDB ID: 7kfz) in complex with STOCK1N-55456 at potential allosteric pocket #2 site obtained from 400 ns MD simulation trajectory **c)** RMSF CA Difference of the ternary complex in complex with STOCK1N-55456 of replica-2 of MD and the ternary complex without ligand. **d)** The ternary structure viewed from two different perspectives, color-coded by RMSF CA Difference from red (highest) to blue (lowest).

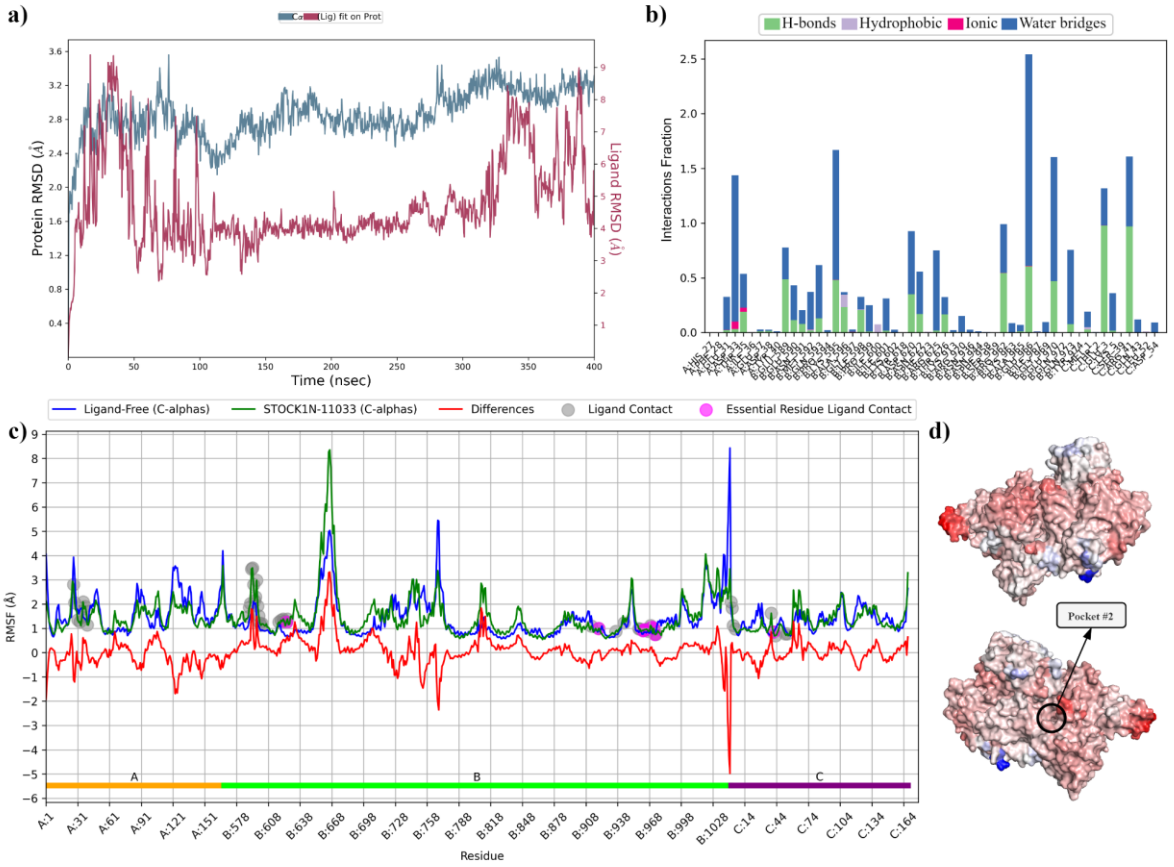

Figure S29. a) Protein and ligand RMSD and **b)** protein-ligand contact histogram of replica-1 of the ternary KRASG13D-SOS1(PDB ID: 7kfz) in complex with STOCK1N-11033 at potential allosteric pocket #2 site obtained from 400 ns MD simulation trajectory **c)** RMSF CA Difference of the ternary complex in complex with STOCK1N-11033 of replica-1 of MD and the ternary complex without ligand. **d)** The ternary structure viewed from two different perspectives, color-coded by RMSF CA Difference from red (highest) to blue (lowest).

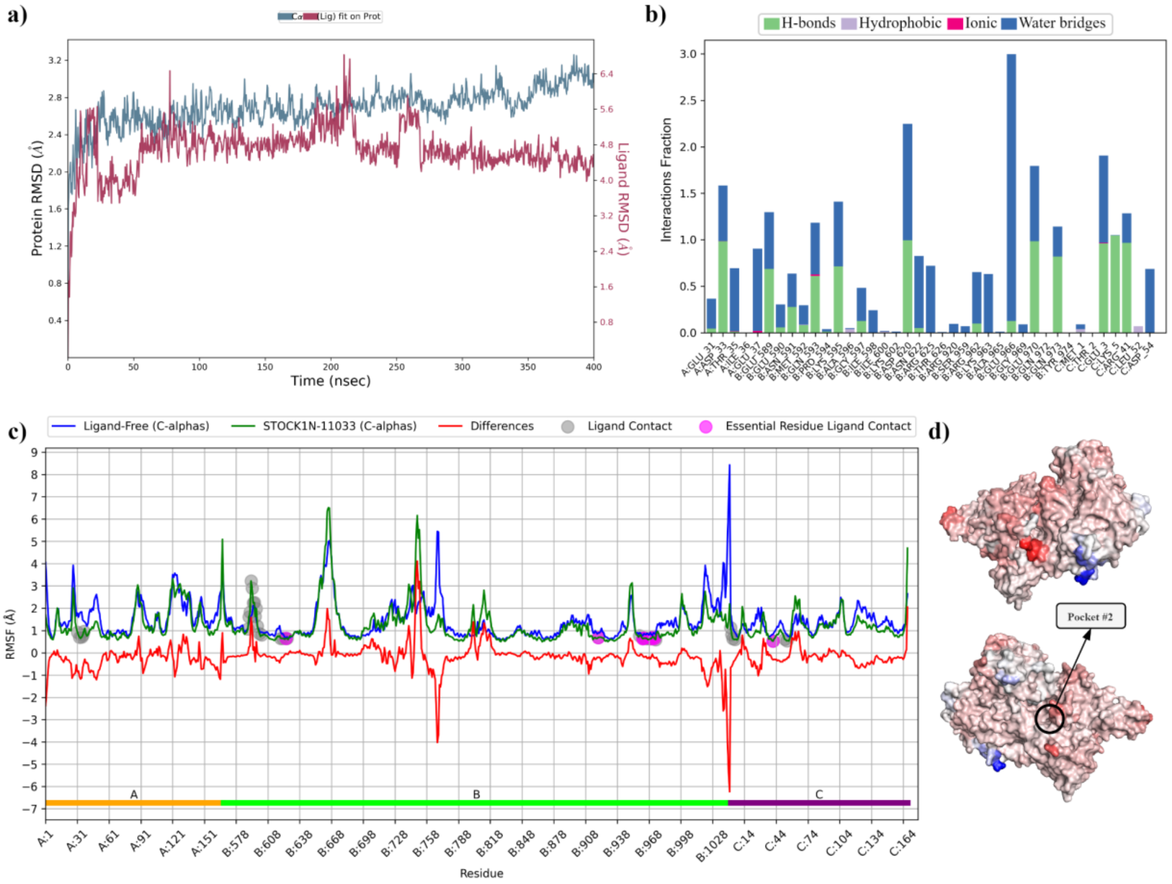

Figure S30. a) Protein and ligand RMSD and **b)** protein-ligand contact histogram of replica-2 of the ternary KRASG13D-SOS1(PDB ID: 7kfz) in complex with STOCK1N-11033 at potential allosteric pocket #2 site obtained from 400 ns MD simulation trajectory **c)** RMSF CA Difference of the ternary complex in complex with STOCK1N-11033 of replica-2 of MD and the ternary complex without ligand. **d)** The ternary structure viewed from two different perspectives, color-coded by RMSF CA Difference from red (highest) to blue (lowest).

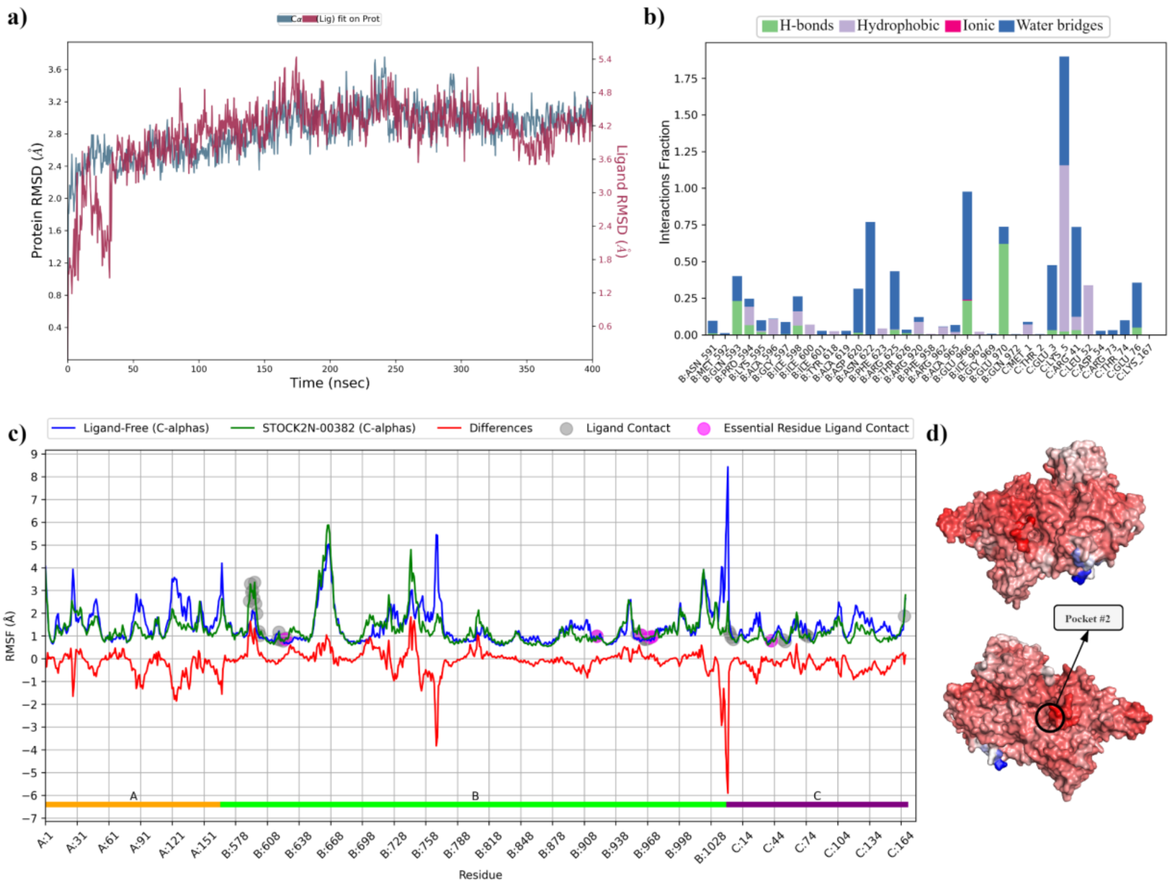

Figure S31. a) Protein and ligand RMSD and **b)** protein-ligand contact histogram of replica-1 of the ternary KRASG13D-SOS1(PDB ID: 7kfz) in complex with STOCK2N-00382 at potential allosteric pocket #2 site obtained from 400 ns MD simulation trajectory **c)** RMSF CA Difference of the ternary complex in complex with STOCK2N-00382 of replica-1 of MD and the ternary complex without ligand. **d)** The ternary structure viewed from two different perspectives, color-coded by RMSF CA Difference from red (highest) to blue (lowest).

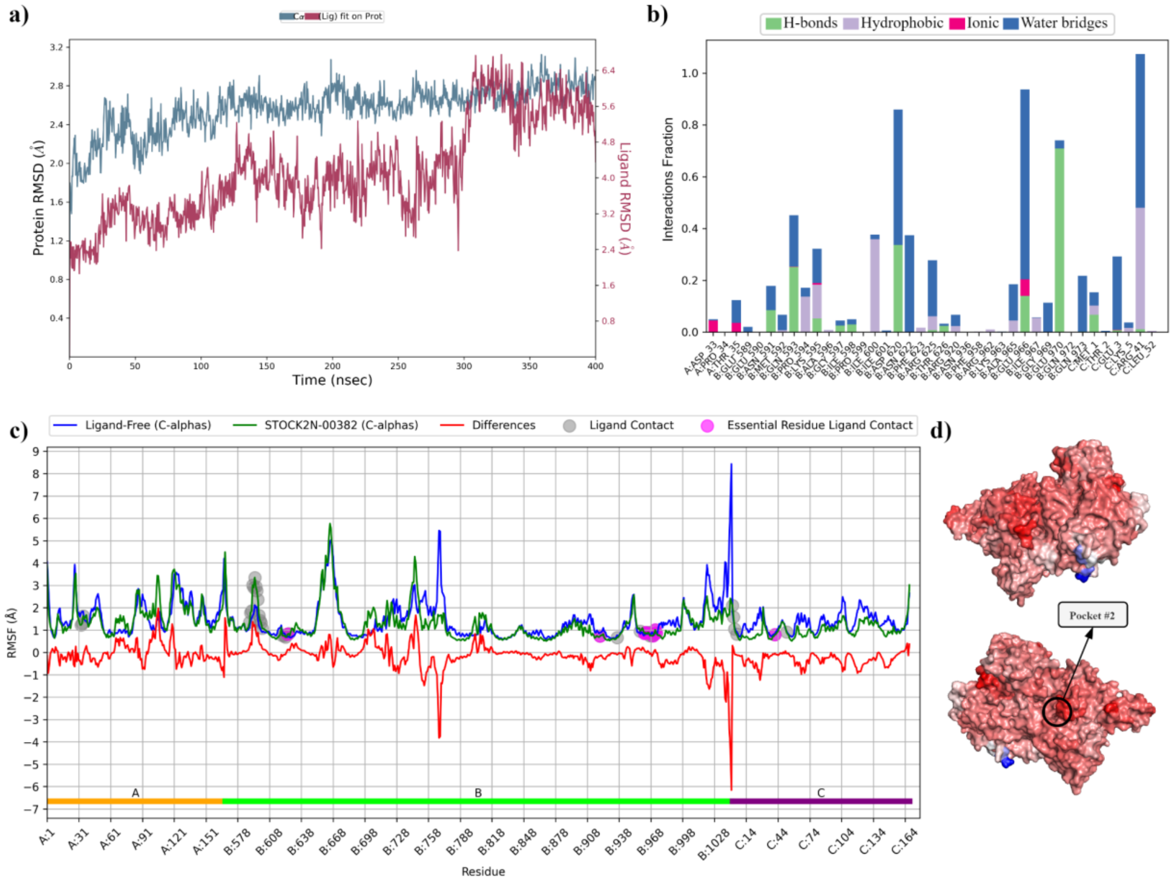

Figure S32. a) Protein and ligand RMSD and **b)** protein-ligand contact histogram of replica-2 of the ternary KRASG13D-SOS1(PDB ID: 7kfz) in complex with STOCK2N-00382 at potential allosteric pocket #2 site obtained from 400 ns MD simulation trajectory **c)** RMSF CA Difference of the ternary complex in complex with STOCK2N-00382 of replica-2 of MD and the ternary complex without ligand. **d)** The ternary structure viewed from two different perspectives, color-coded by RMSF CA Difference from red (highest) to blue (lowest).

3.5. The Distance of R73/N879 and R73/Y884

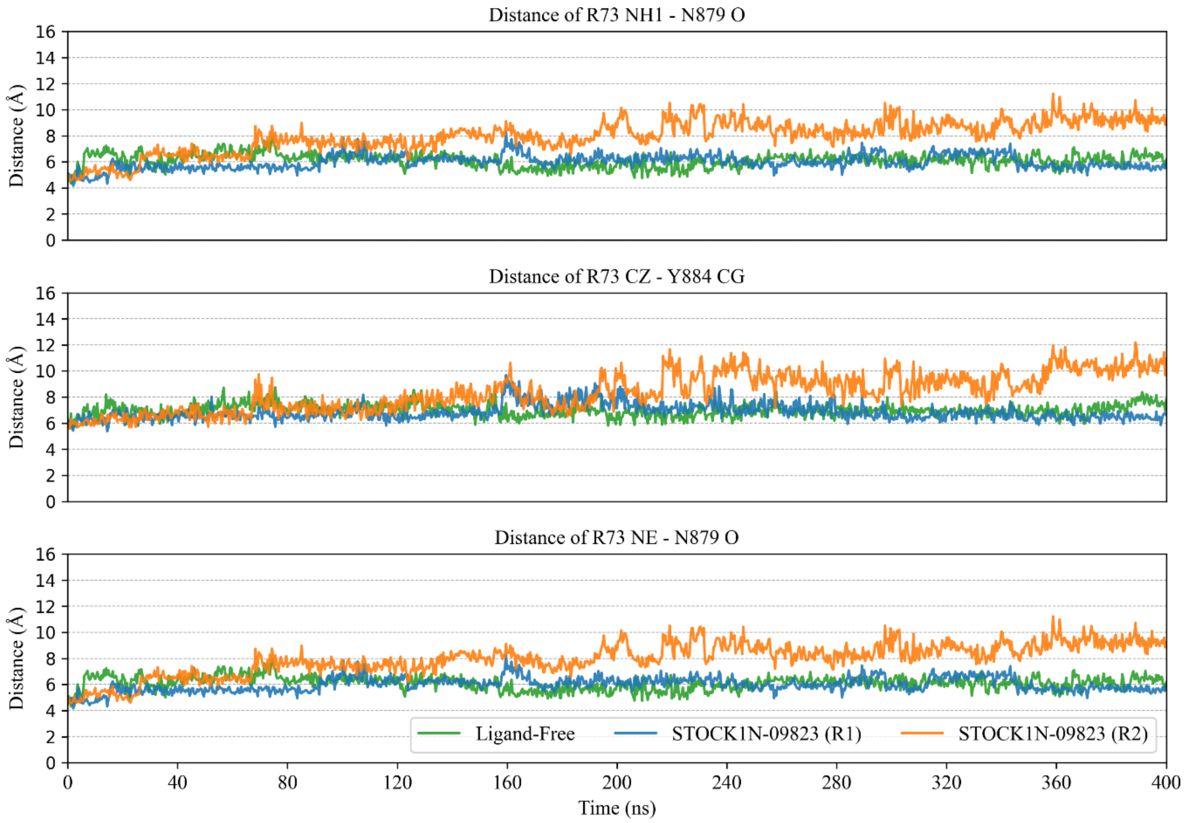

Figure S33. The distance between R73-N879 and R73-Y884 during the MD trajectories.

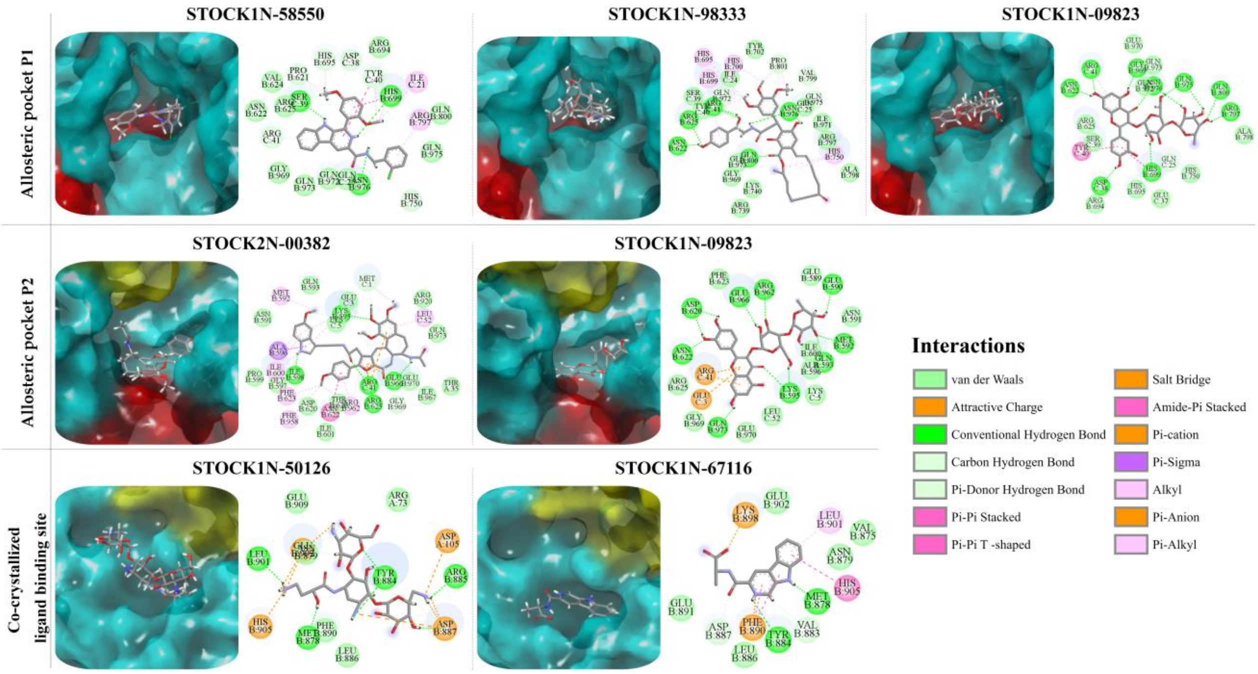

Figure S34. 2-dimensional interaction maps and the binding poses of the hit compounds at the ligand binding site and two putative allosteric sites P1 and P2. In the binding poses of the ligands (in sticks), SOS1, KRAS allosteric and KRAS catalytic are shown in turquoise, red, and yellow surfaces, respectively. The types of interactions in the 2-dimensional maps are color-coded and indicated in the figure.

3.6. ADMET analysis of the compounds

Table S8. Pharmacokinetic and physiochemical properties of the selected hit compounds and SOS1 inhibitors.

Compounds	STOCK1N-98333	STOCK2N-00382	STOCK1N-67116	STOCK1N-58550	STOCK1N-50126	STOCK1N-09823	BAY-293	BI-3406	MRTX0902	Range or recommended values
**molecular weight (g/mol)**	691.8	745.8	283.3	471.9	585.6	610.5	448.6	462.5	388.5	130.0 – 725.0
**PISA**	149.5	219.8	260.9	460.3	0	191.3	244.3	117.7	162.3	0.0 – 450.0
**WPSA**	0	0	0	46.8	0	0	30.8	106.5	0	0.0 – 175.0
**HB donors**	4.0	3.0	2.3	2.0	17.0	9.0	2	2.5	1	0.0 – 6.0
**HB acceptors**	11.7	11.3	4.8	5.0	26.9	20.6	5.5	6.7	7.2	2.0 – 20.0
**QPlogPo/w**	4.5	5.3	2.4	6.3	-8.5	-2.5	5.4	4.7	3.4	−2.0 – 6.5
**QPlogHERG**	-4.1	-3.2	-3.5	-7.5	-6.3	-5.6	-6.8	-5.3	-5.2	concern below −5
**QPPCaco (nm/sec)**	81.7	280	51.8	2315.8	0.003	1.0	1107.3	1557.9	732.6	<25 poor, >500 great
**QPlogBB**	-2.4	-1.5	-1.3	-0.4	-4.9	-4.7	0.2	-0.5	-0.9	−3.0 – 1.2
**QPPMDCK (nm/sec)**	49.6	256.5	25.7	2211.8	0.001	0.3	901.1	3060.2	353.4	<25 poor, >500 great
**%Human Oral Absorption**	61.4	62.7	71.6	100	0	0	100	100	100	>80% is high, <25% is poor
**Rule Of Five**	2	3	0	1	3	3	1	0	0	maximum is 4

PISA: π component of the SASA; WPSA: weakly polar component of the SASA; HB donors/acceptors: number of hydrogen bonds that would be donated/accepted to/from water molecules averaged over several configurations; QPlogPo/w: predicted octanol/water partition coefficient; QPlogHERG: the IC_{50} value for blockage of HERG K^+ channels; QPPCaco: predicted apparent Caco-2 cell (model for the gut-blood barrier) permeability for non-active transport; QPlogBB: predicted brain/blood partition coefficient; QPPMDCK: predicted apparent MDCK cell (model for blood-brain barrier) permeability for non-active transport; % Human Oral Absorption: predicted human oral absorption based on a multiple linear regression model; Rule of Five is the number of violations of Lipinski's rule of five (molecular weight < 500 g/mol, QPlogPo/w < 5, HB donor ≤ 5, HB acceptor ≤ 10).

Table S9. Toxicity analysis of the selected hit compounds and SOS1 inhibitors

**Compounds**	**STOCK1N-98333**	**STOCK2N-00382**	**STOCK1N-67116**	**STOCK1N-58550**	**STOCK1N-50126**	**STOCK1N-09823**	**BAY-293**	**BI-3406**	**MRTX0902**
**AMES Toxicity**	No	No	No	No	No	Yes	Yes	No	No
**Max. Tolerated dose (human)** (log mg/kg/day)	0.71	0.34	0.79	0.47	0.44	0.47	0.78	0.32	-0.12
**Oral Rat Acute Toxicity (LD_{50})** (mol/kg)	2.73	3.07	2.45	2.72	2.48	2.39	2.44	2.70	2.51
**Oral Rat Chronic Toxicity (LOAEL)** (log mg/kg_bw/day)	3.41	2.30	1.93	0.91	7.26	5.23	1.73	1.29	1.40
**Hepatotoxicity**	Yes	No	Yes	Yes	No	No	Yes	Yes	Yes
**Skin Sensitization**	No	No	No	No	No	No	No	No	No
